# Organ–System Predictors of Immune–Related Adverse Events and Their Prognostic Impact in Immune Checkpoint Inhibitors–Treated Cancer Patients: A MENA Retrospective Cohort

**DOI:** 10.3390/cancers18132167

**Published:** 2026-07-06

**Authors:** Ali Awada, Ali Tarhini, Abbas Hammoud, Mohammad Kassem, Joe Rizkallah, Mohammad Al Hajjar, Ali Dakik, Michael Romanos, Sary Faraj, Duha Awada, Lara Soueid, Razane Wehbe, Karim Kalout, Nicole Charbel, Firas Kreidieh

**Affiliations:** 1 Division of Hematology and Oncology, Department of Internal Medicine, American University of Beirut, Beirut P.O. Box 11-0236, Lebanon; ama221@mail.aub.edu (A.A.); at110@aub.edu.lb (A.T.); ahh85@mail.aub.edu (A.H.); mak121@mail.aub.edu (M.K.); mta38@aub.edu.lb (M.A.H.); amd41@mail.aub.edu (A.D.); mbr12@mail.aub.edu (M.R.); duha.awada@std.balamand.edu.lb (D.A.); lxs03@mail.aub.edu (L.S.); riw04@mail.aub.edu (R.W.); nc47@aub.edu.lb (N.C.); 2 Department of Diagnostic Radiology, American University of Beirut, Beirut P.O. Box 11-0236, Lebanon; jr56@aub.edu.lb

**Keywords:** immune checkpoint inhibitors, immune-related adverse events, cancer immunotherapy, overall survival, progression-free survival, objective response rate, predictors

## Abstract

**Background:** Immune checkpoint inhibitors (ICIs) have transformed cancer treatment but are associated with immune-related adverse events (irAEs) and variable clinical outcomes. Clinical predictors of organ-specific irAEs remain indeterminate, particularly in real-world populations. **Methods:** We conducted a retrospective single-center cohort study including 751 adult patients with solid tumors treated with ICIs between 2018 and 2025. Clinical, demographic, and treatment-related variables were analyzed. Multivariable logistic regression identified predictors of irAEs, while associations with objective response rate (ORR), progression-free survival (PFS), and overall survival (OS) were assessed using logistic and Cox regression models. **Results:** The most frequent irAEs were endocrine (9.9%), dermatologic (9.1%), gastrointestinal (7.6%), and pulmonary (4.7%). Female sex independently predicted endocrine (aOR 1.98, *p* = 0.007) and rheumatologic irAEs (aOR 4.06, *p* = 0.007). Combination immunotherapy was associated with increased dermatologic (aOR 2.66, *p* = 0.013) and gastrointestinal irAEs (aOR 2.65, *p* = 0.016), while concurrent radiotherapy predicted gastrointestinal toxicity (aOR 1.82, *p* = 0.044). Atezolizumab was associated with higher pulmonary irAE risk (aOR 2.97, *p* = 0.048). Endocrine, dermatologic, gastrointestinal, and pulmonary irAEs were independently associated with improved ORR (OR range: 2.53–4.30, all *p* < 0.01). **Conclusions:** Organ-specific irAEs exhibit distinct clinical predictors and differential prognostic implications in patients receiving ICIs. Select irAEs are associated with improved treatment response and disease control, yet our results should be regarded as hypothesis-generating requiring further investigation.

## Simple Summary

Immune checkpoint inhibitors have improved outcomes across multiple malignancies but are frequently complicated by immune-related adverse events, the organ-specific predictors of which remain incompletely defined. In this retrospective cohort of 751 patients treated with Immune checkpoint inhibitors in Lebanon, we evaluated clinical and treatment-related predictors of organ-specific irAEs and their association with treatment outcomes. Female sex was independently associated with endocrine and rheumatologic irAEs, whereas combination immunotherapy and concurrent radiotherapy were associated with increased dermatologic and gastrointestinal toxicity. Endocrine, dermatologic, gastrointestinal, and pulmonary irAEs were each independently associated with improved objective response. As such associations are inherently susceptible to immortal time bias, these findings should be regarded as hypothesis-generating and warrant prospective validation before informing clinical decision-making.

## 1. Introduction

Immunotherapy is a modality used in the treatment of cancer that has reshaped the management plans for many malignancies [1]. It enhances the role of the immune system in detecting and destroying cancerous cells through many mechanisms [2]. Among immunotherapeutic strategies are the immune checkpoint inhibitors (ICIs) which has led to a notable progression in the management of several diverse cancer types, including melanoma, renal cell carcinoma, bladder cancer, and non-small cell lung cancer [3]. ICIs work by blocking the co-inhibitory receptors expressed on surface T cells such as programmed cell death protein-1 (PD-1), programmed death-ligand 1 (PD-L1), and cytotoxic T-lymphocyte-associated protein 4 (CTLA-4), thereby activating these cytotoxic T cells and restoring their antitumor activity [4]. After the widespread integration of ICIs into treatment plans, the use of combination regimens has led to meaningful gains in overall survival and progression-free survival rates for many subsets of patients across different malignancies [5]. The combination of PD-1 and CTLA-4 inhibitors in the setting of metastatic melanoma have shown higher response rates, prolonged progression-free survival, and 5-year overall survival rates approaching 40–50% compared to survival rates below 10% before the introduction of immunotherapy [6].

One major drawback of ICIs is the immune-related adverse events (irAEs). These events correspond to a spectrum of toxicities that arise due to the nonspecific activation of immune cells against normal tissues as well as loss of immune tolerance which leads to inflammatory damage of healthy organs [7]. Multiple mechanisms to explain irAEs have been proposed which include activation of autoreactive T and B cells leading to the production of autoantibodies, as well as the mediation of inflammatory cytokines such as interleukins [8]. IrAEs may represent a treatment-limiting barrier for several malignancies based on their grade on presentation [9]. The toxicities are evaluated using the Common Terminology Criteria for Adverse Events (CTCAE) which grades the toxicity from grade 1 to grade 5, where higher grades indicate increasing clinical severity [10].

The overall incidence of irAEs of any grade is reported to range from 15 to 90% for single agents, with approximately 13% presenting as high-grade toxicity which require immunosuppression and immunotherapy withdrawal [10]. However, this incidence differs between the different types of ICI, with more irAEs seen in anti-CTLA-4 inhibitors compared to anti-PD-1 and anti-PD-L1 inhibitors [11].

IrAEs can affect any organ system with different timing of appearance, with skin manifestations appearing the earliest at 2–3 weeks following the induction of first dose of ICI [12,13]. The most commonly organ systems affected include the skin, gastrointestinal, pulmonary, and endocrine systems which present as rash and pruritis, colitis, hepatitis, pneumonitis, thyroiditis and adrenal insufficiency respectively [12,14,15,16].

There exist potential organ-specific biomarkers that might predict a patient’s risk of developing irAEs. However, clinical predictors of organ-specific irAEs remain indeterminate [17]. In addition, several demographic factors, disease-related factors, and treatment-related factors were described as potential predictors of overall risk of developing irAEs. To begin with, the sex of the individual remains a debatable predictor of irAEs despite findings suggestive of higher incidence of irAEs in women compared to men [13]. Also, the use of combination regimens of ICIs with chemotherapy showed increased incidence of adverse events, although the efficacy of the immunotherapy treatment was much better [13]. In addition, patients with pre-existing autoimmune diseases have a relatively higher risk of irAEs [13]. Patients who were previously treated with statins or received pre-ICI systemic corticosteroids had a higher risk of developing irAEs [18]. Furthermore, patients with comorbidities such as myocardial infarction, heart failure, and renal disease have also an increased risk of developing irAEs [19].

While the factors described are beneficial to predict the overall risk of developing irAEs in patients receiving immunotherapy, the clinical predictors of organ-specific irAEs remain incompletely characterized, particularly in real-world patient populations. Therefore, the present study aims to evaluate these clinical predictors that are associated with the development of organ-specific irAEs in cancer patients receiving ICIs in an attempt to improve risk stratification, targeted monitoring, and developing early intervention strategies for these patients.

## 2. Methodology

This retrospective, single-center observational study aimed to evaluate clinical predictors of organ-specific immune-related adverse events (irAEs) in cancer patients receiving immune checkpoint inhibitors. The sample consisted of 751 adults (aged 18 years or older) diagnosed with any type of solid malignancy, who received ICIs as part of their treatment regimen at the American University of Beirut Medical Center between January 2018 and January 2025. The ICIs used included Ipilimumab, Nivolumab, Pembrolizumab, Atezolizumab, Avelumab, Durvalumab, or any combination of these drugs.

Parameters collected included demographic characteristics, clinical status, cancer-related variables, and treatment characteristics. Demographic and baseline clinical data included age, sex, body mass index (BMI), smoking history, Charlson Comorbidity Index (CCI), Eastern Cooperative Oncology Group (ECOG) performance status, and the presence of preexisting comorbidities including diabetes mellitus, baseline thyroid abnormalities, and preexisting lung diseases. Cancer-related variables included tumor type, presence of metastases, and other disease characteristics. Treatment-related variables included prior history or concurrent administration chemotherapy or radiotherapy, as well as immunotherapy regimen characteristics, including ICI agents, whether administered alone or in combination.

Detailed data on irAEs were also collected, including the affected organ system, severity, onset, management strategies, and clinical outcomes. IrAEs were graded using the Common Terminology Criteria for Adverse Events (CTCAE), version 5.0.

IrAEs were ascertained through structured review of electronic medical records, including oncology clinic notes, hospitalization notes, and relevant laboratory, imaging, and pathology reports, by the oncology team. Attribution of an adverse event to ICI therapy was based on the treating physician’s clinical judgment. Exclusion of alternative etiologies was achieved through standard clinical evaluation, where competing causes were evaluated as part of routine clinical care before an event was classified as immune-related. However, since the ascertainment relied on retrospective documentation, residual misclassification of irAEs cannot be completely excluded.

The primary outcome of this study was the identification of independent clinical predictors of organ-specific irAEs following initiation of ICI therapy, with each organ-specific irAE treated as an independent binary outcome. Multivariate logistic regression models were constructed for seven systems; neurologic and circulatory irAEs were excluded from multivariate modeling due to insufficient event counts (*n* = 7 and *n* = 3, respectively). Secondary outcomes included overall response rate (ORR), defined as complete or partial response per treating physician assessment; progression-free survival (PFS), defined as time from ICI initiation to disease progression or recurrence; and overall survival (OS), defined as time from ICI initiation to death from any cause. RECIST criteria was used to measure the treatment response. Patients without an event were censored at the date of last follow-up. For survival analyses, four irAE systems were evaluable: endocrine, dermatologic, gastrointestinal, and pulmonary as renal and systemic irAEs were additionally excluded due to insufficient event counts for Cox modeling.

For each organ-specific irAE outcome, all candidate baseline predictors were first screened using univariate binary logistic regression. Predictors were then entered into organ-specific multivariate binary logistic regression models, with the number of predictors constrained by an events-per-predictor ratio of 8:1 to avoid overfitting. Predictors were ranked by univariate *p*-value and selected in descending order of significance up to the maximum allowable number per model. Where standard logistic regression produced unstable estimates defined as non-convergence, any coefficient being missing or non-finite, or any non-intercept coefficient exceeding an absolute value of 10, then Firth penalized logistic regression was applied as a fallback. Firth’s method adds a penalty term to the likelihood function that prevents infinite coefficient estimates in the presence of sparse predictor categories or quasi-complete separation, producing finite and less biased estimates. The modeling approach applied for each irAE system is reported alongside the results. Reference categories were defined as “No” for all binary variables, “Male” for sex, NSCLC for primary diagnosis, Pembrolizumab for immunotherapy type, and the most frequent category for remaining multi-level factors. Odds ratios with 95% confidence intervals derived from profile likelihood where possible and Wald-based otherwise are reported.

Kaplan-Meier curves were generated for OS and PFS stratified by irAE status for each evaluable organ system, with between-group differences assessed using the log-rank test. Univariate Cox proportional hazards regression was performed to screen endocrine, dermatologic, gastrointestinal, and pulmonary irAEs alongside all baseline covariates—as predictors of OS and PFS across the full cohort. Multivariate Cox proportional hazards regression models were then constructed for both OS and PFS, with all four irAE systems forced into each model alongside baseline confounders—specifically age, sex, BMI, CCI, and ECOG performance status—and any additional predictor demonstrating a univariate association at *p* < 0.15.

ORR was analyzed using multivariate binary logistic regression, with all four major irAE subtypes simultaneously included alongside baseline confounders demonstrating a univariate association at *p* < 0.15.

The proportion of missing data varied across key analytical variables. ECOG performance status was missing in 236 patients (31.4%), disease response status in 68 patients (9.1%), disease stage in 24 patients (3.2%), BMI in 9 patients (1.2%), and CCI in 6 patients (0.8%). All analyses used complete-case analysis, with the available sample size reported per model. Because complete-case analysis required non-missing values for every covariate entered into a given model, analytic sample sizes for adjusted multivariable models are necessarily smaller than the full cohort of 751 patients, reflecting the cumulative effect of missingness across all included covariates rather than missingness in any single variable; the sample size and number of events are reported for each model presented.

Given the large number of predictors and outcomes evaluated across multiple organ systems, all analyses are considered exploratory and hypothesis-generating; no correction for multiple comparisons was applied. All statistical analyses were performed in R. Statistical significance was defined at a two-sided *p*-value of 0.05. This study was approved by the Institutional Review Board at the American University of Beirut, with a waiver of informed consent.

## 3. Results

### 3.1. Baseline Characteristics

The cohort included 751 patients (mean age 68.07 ± 12.93 years; BMI 26.31 ± 4.84 kg/m^2^). Most patients were male (65%) with a high comorbidity burden (median Charlson Comorbidity Index 8.0 [IQR 6.0–10.0]). Non-small cell lung cancer (NSCLC) was the predominant diagnosis, accounting for 45% of cases. Most patients had stage IV disease (75%) and good performance status (ECOG 0–1: 94%). Concerning treatment regimens, 56% received pembrolizumab, 63% underwent concurrent chemotherapy, and 48% received concurrent radiotherapy (Table 1). The distribution of immune-related adverse events (irAEs) is detailed in Table 2, with endocrine (9.9%), dermatologic (9.1%), gastrointestinal (7.6%), and pulmonary (4.7%) toxicities being most frequent.

Each organ system was treated as an independent binary outcome; patients who developed irAEs in more than one organ system were counted in each relevant category. Of 751 patients, 260 (34.6%) experienced at least one irAE of any type.

### 3.2. System Predictors

Multivariable analysis using Firth penalized logistic regression identified independent predictors of irAEs; univariate results are provided in the Supplementary Materials S1. In the model for endocrine irAEs, female sex was the only significant predictor (aOR 1.98, *p* = 0.007). For dermatologic irAEs, significant predictors included combination immunotherapy (aOR 2.66, *p* = 0.013), previous targeted therapy (aOR 0.08, *p* = 0.007), and gastrointestinal diagnosis (aOR 2.74, *p* = 0.028). Gastrointestinal irAEs showed notable associations with combination therapy (aOR 2.65, *p* = 0.016), prior adverse reactions to immunotherapy (aOR 7.56, *p* = 0.023), age (aOR 0.87, *p* = 0.015), and concurrent radiotherapy (aOR 1.82, *p* = 0.044). Pulmonary irAEs were associated with atezolizumab (vs pembrolizumab) (aOR 2.97, *p* = 0.048). Bladder cancer (vs NSCLC) predicted renal (aOR 4.91, 95% CI 1.61–15.01, *p* = 0.004) and systemic irAEs (aOR 7.11 (95% CI 1.35–43.71), *p* = 0.022). Female sex was also a significant predictor for rheumatologic irAEs (aOR 4.06, *p* = 0.007). Other variables, such as concurrent or prior chemotherapy and various alternative diagnoses, did not reach statistical significance across these systems. All results of multivariable logistic regression of predictors per irAE system are summarized in Table 3.

### 3.3. Efficacy and Survival Outcomes

#### 3.3.1. Overall Response Rate (ORR)

In the multivariable analysis, several factors emerged as significant independent predictors of ORR (Table 4). Endocrine (OR 2.69, 95% CI 1.54–4.77, *p* < 0.001), dermatologic (OR 4.07, 95% CI 2.25–7.58, *p* < 0.001), gastrointestinal (OR 2.53, 95% CI 1.34–4.88, *p* = 0.005), and pulmonary irAEs (OR 4.30, 95% CI 1.89–10.38, *p* < 0.001) were associated with higher response rates. Lower ORR was associated with higher Charlson Comorbidity Index (OR 0.85 per point, 95% CI 0.79–0.91, *p* < 0.001), prior steroid use (OR 0.62, 95% CI 0.39–0.97, *p* = 0.038), and prior chemotherapy (OR 0.61, *p* = 0.047). Other variables, such as sex and immunotherapy type, did not reach significance in the multivariable response model.

#### 3.3.2. Progression-Free Survival (PFS)

In univariate analysis, bladder cancer, melanoma, renal cell carcinoma (HR 0.25, *p* = 0.003 & HR 0.22, *p* = 0.01 & HR 0.26, *p* = 0.004), and primary tumor surgery (HR 0.51, *p* < 0.001) were associated with improved PFS, whereas concurrent chemotherapy was associated with worse PFS (HR 1.98, *p* < 0.001). Pulmonary and Gastrointestinal irAEs were not significantly associated with PFS (*p* = 0.93 and *p* = 0.92, respectively). When adjusted for cofounders in the multivariable analysis, only concurrent chemotherapy (HR 2.30, *p* = 0.003) remained associated with worse PFS (Supplementary Materials S2). Additionally, Kaplan–Meier curves of PFS stratified by irAEs are presented in the Supplementary Materials S3.

#### 3.3.3. Overall Survival

In univariate analysis, higher Charlson Comorbidity Index (HR 1.14, *p* < 0.001), ECOG 2–4 (HR 2.46, *p* < 0.001), prior steroid use (HR 1.41, *p* = 0.013), using combination Immunotherapy (HR 1.52, *p* = 0.043) and prior immunotherapy (HR 2.53, *p* = 0.025) were associated with worse OS. Primary tumor surgery was associated with improved OS (HR 0.70, *p* = 0.018). Conversely, irAEs were not significantly associated with OS, including gastrointestinal (HR 0.98, *p* = 0.927) and endocrine irAEs (*p* = 0.213). When adjusted for cofounders in the multivariable analysis, higher Charlson Comorbidity Index (HR 1.19, *p* < 0.001), ECOG 2–4 (HR 1.88, *p* < 0.024), and using combination Immunotherapy (HR 1.78, *p* = 0.023) remained significant predictors of worse OS (Supplementary Materials S2). Kaplan–Meier curves of OS stratified by irAEs are presented in the Supplementary Materials S3.

## 4. Discussion

In this large retrospective cohort of 751 patients receiving immune checkpoint inhibitors (ICIs), we identified distinct clinical predictors and prognostic associations for organ-specific immune-related adverse events (irAEs). Endocrine and rheumatologic irAEs were more common in female patients, whereas combination immunotherapy was associated with increased dermatologic and gastrointestinal toxicities. Concurrent radiotherapy and prior immune-related toxicity further increased the likelihood of gastrointestinal irAEs, while atezolizumab use was associated with higher pulmonary toxicity risk. Importantly, endocrine, dermatologic, gastrointestinal, and pulmonary irAEs were associated with significantly improved ORR. No significant association between irAEs and PFS and OS. These findings highlight the heterogeneous biological and prognostic nature of irAEs and support the hypothesis that immune toxicity may represent a dynamic marker of immune activation rather than a uniform treatment complication. However, because irAE ascertainment is conditional on survival and continued treatment, the observed ORR associations may partly reflect immortal time bias rather than a causal effect, as discussed in the Limitations.

The overall incidence and distribution of irAEs in our cohort were consistent with previously reported real-world data. Endocrine, dermatologic, and gastrointestinal toxicities represented the most common irAE categories, mirroring findings from large meta-analyses and pharmacovigilance studies demonstrating that skin, endocrine, and gastrointestinal systems are among the most frequently affected organs during PD-1/PD-L1 and CTLA-4 blockade. The observed irAE rate of 34.6% also aligns with contemporary studies reporting overall irAE incidences ranging between 30% and 45%, particularly in cohorts predominantly treated with anti–PD-1 therapy [9,20].

Female sex independently predicted endocrine and rheumatologic irAEs in our analysis. This observation is biologically plausible and consistent with previous evidence suggesting sex-related differences in immune regulation and autoimmunity. One speculative explanation is that women generally exhibit stronger humoral and cellular immune responses, potentially increasing susceptibility to autoimmune toxicities during immune checkpoint blockade. Recent reviews have similarly identified female sex as a risk factor for thyroid dysfunction and rheumatologic irAEs during ICI therapy [21,22,23]. Hormonal influences, differences in T-cell activation, and higher baseline prevalence of autoimmune predisposition among women may contribute to this phenomenon.

Combination immunotherapy emerged as a major predictor of dermatologic and gastrointestinal irAEs. This finding is concordant with established evidence demonstrating that dual checkpoint inhibition produces broader immune activation and substantially increases toxicity risk compared with monotherapy [23]. CTLA-4 inhibition, in particular, has been associated with immune-mediated colitis and cutaneous toxicities, possibly related to enhanced T-cell priming and reduced peripheral immune tolerance. The significantly increased risk of gastrointestinal irAEs observed with prior irAE history also suggests that certain patients may possess an intrinsic predisposition toward immune dysregulation during checkpoint inhibition. Such susceptibility may reflect underlying host immunogenetic characteristics, cytokine profiles, microbiome composition, or occult autoimmune tendencies that remain incompletely characterized [24,25].

Our multivariable model also revealed critical, system-specific associations tied directly to primary tumor biology and previous clinical exposures. A key finding was that a primary diagnosis of bladder cancer was associated with a nearly five-fold increase in the odds of developing renal irAEs and a striking seven-fold increase in systemic irAEs. This localized urinary tract vulnerability may be driven by tissue-specific antigens shared between the bladder urothelium and renal parenchymal cells, or direct organ-specific immune niches, drawing accelerated T-cell clones directly to these organs [26,27]. Conversely, prior targeted therapy was associated with a markedly lower likelihood of dermatologic irAEs, suggesting that sequential small-molecule inhibitors (such as anti-angiogenic or MAPK-pathway TKIs) may modulate the baseline cutaneous microenvironment, reducing the inflammation cascades required to manifest severe checkpoint-inhibitor skin reactions [28]. We also observed that a gastrointestinal diagnosis predicted higher dermatologic toxicities, whereas a hepatopancreatobiliary diagnosis exerted a strong protective effect against gastrointestinal irAEs. These complex variations underline the fact that irAEs are not merely random systemic events, but localized phenomena shaped by baseline primary tumor characteristics and distinct organ-specific immune settings [29]. We considered stratified multivariable analyses by tumor type (NSCLC, melanoma, and urothelial carcinoma) but did not perform them in the present analysis because of inadequate sample size: even the largest non-NSCLC subgroups (melanoma, *n* = 51; bladder cancer, *n* = 65) would yield very few events once subdivided across nine organ-specific irAE categories, risking unstable, non-convergent, or uninterpretable models.

Concurrent radiotherapy independently predicted gastrointestinal irAEs in our cohort. Although the mechanistic relationship remains incompletely understood, radiotherapy is known to induce inflammatory cytokine release, antigen presentation, and immune priming, potentially augmenting immune-mediated tissue injury when combined with ICIs [30]. Previous studies have suggested synergistic immune activation between radiation and checkpoint blockade, including enhancement of both antitumor effects and toxicity profiles [30,31]. The gastrointestinal tract may be particularly vulnerable because of its immunologically active mucosal environment and microbiome-dependent immune regulation [32].

Atezolizumab was associated with a significantly higher likelihood of pulmonary irAEs compared with pembrolizumab. Although previous literature has demonstrated variable pneumonitis rates among PD-1 and PD-L1 inhibitors, pulmonary toxicity remains one of the most clinically significant irAEs because of its potential severity and mortality risk [33]. Differences in pneumonitis incidence between ICIs may reflect variations in immune signaling pathways, patient selection, prior thoracic irradiation exposure, or underlying pulmonary vulnerability. Given the potentially fatal nature of immune-related pneumonitis, these findings may have practical implications for surveillance strategies in high-risk populations.

An important finding of this study was the strong association between several irAE subtypes and improved treatment response. Endocrine, dermatologic, gastrointestinal, and pulmonary irAEs all independently predicted superior ORR. These results support the growing body of evidence suggesting that irAEs may serve as surrogate markers of robust immune activation and enhanced antitumor activity. Mechanistically, both antitumor efficacy and irAEs likely arise from overlapping immune activation pathways involving cytotoxic T-cell expansion, cytokine release, and loss of peripheral immune tolerance. Several studies in melanoma, NSCLC, and renal cell carcinoma have similarly demonstrated improved response rates and survival outcomes among patients developing irAEs, particularly dermatologic and endocrine toxicities [34,35,36,37]. However, because development of an irAE necessarily presupposes that a patient has survived, and remained on treatment, long enough for the toxicity to manifest and be ascertained, this association is potentially susceptible to immortal time bias rather than reflecting a purely causal link between immune toxicity and antitumor efficacy; this issue is addressed further in the Limitations section below.

In contrast to the strong ORR associations, no irAE subtype was independently associated with PFS or OS in adjusted Cox models. Although gastrointestinal irAE showed a numerically lower hazard of progression (HR 0.49, 95% CI 0.23–1.03, *p* = 0.062), this did not reach statistical significance, and the corresponding Kaplan–Meier comparison was also non-significant. This pattern is expected since irAEs ascertainment is conditional on survival and continued treatment exposure, because response is assessed earlier and is more vulnerable to immortal time bias than time-to-event endpoints that begin at treatment initiation. It may also reflect treatment interruption, immunosuppressive management of severe toxicity, and the influence of subsequent lines of therapy. The absence of a significant survival benefit is consistent with contemporary analyses demonstrating that the prognostic implications of irAEs vary substantially according to organ system, severity, timing, and cancer type [34,38].

Notably, pulmonary and systemic irAEs were not associated with improved PFS in our cohort. This finding may reflect the clinically disruptive nature of severe pulmonary toxicity, which frequently necessitates treatment interruption, high-dose corticosteroids, hospitalization, or permanent discontinuation of ICIs. Previous studies have shown that mild irAEs often correlate with favorable outcomes, whereas severe toxicities may paradoxically associate with poorer survival because of treatment discontinuation and increased morbidity [38]. Therefore, the prognostic implications of irAEs likely depend not only on the affected organ system but also on toxicity severity and management requirements.

**Clinical Implications.** Our findings have several important clinical implications. First, identification of system-specific predictors may generate hypothesis for pretreatment risk stratification and facilitate personalized toxicity monitoring. Patients receiving combination immunotherapy, women, patients with prior irAEs, and those undergoing concurrent radiotherapy may benefit from closer surveillance and earlier multidisciplinary intervention. Second, the association between irAEs and improved ORR reinforces the concept that immune toxicity may represent a clinically meaningful biomarker of treatment activity. However, these associations should not encourage intentional continuation of therapy during severe toxicity, as high-grade irAEs may still produce substantial morbidity and compromise long-term outcomes. Third, the observed heterogeneity among organ-specific irAEs suggests that future predictive models should analyze irAEs individually rather than collectively, as combining all toxicities into a single category may obscure clinically relevant biological differences.

**Limitations.** This study has several limitations. Its retrospective single-center design introduces potential selection bias, missing data, and residual confounding despite multivariable adjustment. The heterogeneous cancer population and treatment regimens may also limit generalizability and complicate interpretation of organ-specific associations. Formal diagnostic testing of the proportional hazards’ assumption (e.g., Schoenfeld residuals) was not performed for the multivariable Cox models, which is acknowledged as an additional methodological limitation. Because development of an irAE requires that a patient survive, and remain on ICI therapy, long enough for the toxicity to occur and be documented, this approach is inherently susceptible to immortal time bias. The same logic applies to ORR, since restaging itself requires sufficient survival and continued treatment exposure. Consequently, the observed associations between irAEs and improved ORR and PFS should be interpreted with caution and may partly reflect survival-dependent ascertainment of irAEs rather than a causal protective effect of immune toxicity. Hence, our findings should be regarded as hypothesis-generating, pending confirmation with such methods in future, prospectively designed studies. Additionally, molecular biomarkers, autoantibody profiles, cytokine levels, microbiome data, and genomic predictors were unavailable, limiting mechanistic interpretation. The relatively small number of certain irAE subtypes also reduced statistical power for less common toxicities. Finally, grading severity and management strategies for irAEs were not comprehensively incorporated into outcome analyses.

Importantly, this study provides some of the first large-scale real-world evidence evaluating organ-specific irAE predictors within a Middle East and North Africa (MENA) cohort. Given that predominant landmark clinical trials heavily reflect Western demographics, demonstrating that overall toxicity rates (34.6%) and classic risk factors (such as female sex and combination therapy) remain consistent in a Middle Eastern population represents a crucial step toward validating global immuno-oncology paradigms across diverse ethnicities.

**Figure 1:**
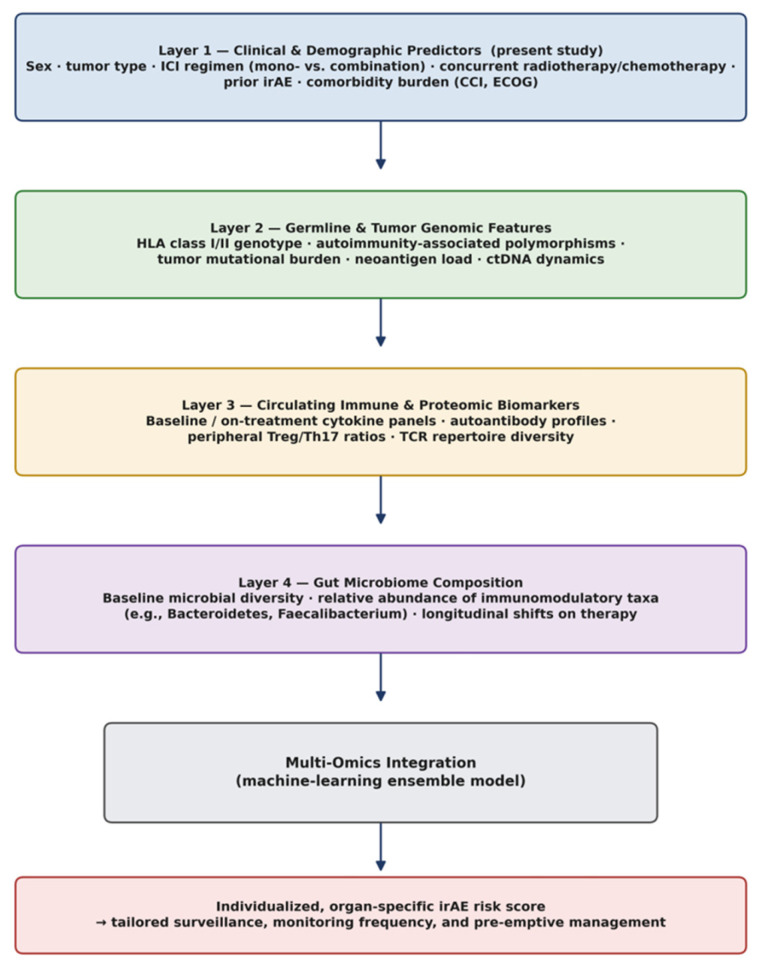
Conceptual multi-omics framework for organ-specific immune-related adverse event (irAE) risk prediction.

Future studies should focus on prospective multicenter validation of organ-specific irAE predictors and integration of clinical, molecular, immunologic, and microbiome-based biomarkers into predictive models. Advanced approaches incorporating artificial intelligence and longitudinal biomarker profiling may further improve individualized toxicity prediction and monitoring [39,40]. Additional research is also needed to clarify the biological mechanisms underlying differential prognostic effects among specific irAE subtypes and to determine whether toxicity-guided treatment adaptation strategies may optimize therapeutic outcomes while minimizing morbidity. For the above reasons, we propose a conceptual multi-omics framework (Figure 1) in which the clinical predictors identified in this study are layered with germline and tumor genomic features (e.g., HLA genotype, autoimmunity-associated polymorphisms, tumor mutational burden), circulating immune and proteomic biomarkers (cytokine panels, autoantibody profiles, peripheral immune cell subsets, T-cell receptor repertoire diversity), and gut microbiome composition, integrated through a machine-learning approach to generate an individualized, organ-specific irAE risk score. This framework illustrates a pathway by which the clinical predictors identified here could be combined with these emerging biomarker classes in future prospective work, moving irAE risk prediction from population-level clinical risk factors toward genuinely personalized surveillance strategies.

## 5. Conclusions

In this large real-world cohort, organ-specific irAEs demonstrated distinct clinical predictors and heterogeneous associations with treatment outcomes. Female sex, combination immunotherapy, prior immune toxicity, and concurrent radiotherapy emerged as key determinants of specific irAE subtypes. Importantly, endocrine, dermatologic, gastrointestinal, and pulmonary irAEs were associated with improved objective response, whereas no irAE subtype was independently associated with progression-free or overall survival after adjustment. These findings support associations between organ-specific irAEs and clinical outcomes, however, our retrospective design does not validate irAEs as biomarkers of immune activation, and the observed associations should be regarded as hypothesis-generating. This highlights the need to adopt organ-specific frameworks in both clinical practice and research. Prospective multicenter studies incorporating molecular, immunologic, and microbiome-based biomarkers are warranted to refine predictive models and optimize the balance between therapeutic efficacy and toxicity in immuno-oncology.

## Figures and Tables

**Table 1:** Baseline Demographic and Clinical Characteristics.

Characteristic	*n* = 751 ^1^
Age, years	68.07 ± 12.93
BMI, kg/m^2^	26.31 ± 4.84
Charlson Comorbidity Index	8.00 [6.00, 10.00]
Number of cycles	14.37 ± 16.59
Treatment duration, months	12.69 ± 15.83
Overall survival, months	23.33 ± 22.25
Progression-free survival, months	20.47 ± 20.55
Sex	
Male	485 (65%)
Female	266 (35%)
Diagnosis	
Bladder Cancers	65 (8.7%)
Gastrointestinal	68 (9.1%)
Hepatobiliary	51 (6.8%)
Melanoma	51 (6.8%)
NSCLC	337 (45%)
Others	123 (16%)
Renal Cell Carcinoma	56 (7.5%)
Disease stage	
Stage < IV	180 (25%)
Stage IV	547 (75%)
ECOG performance status	
0–1 Active	486 (94%)
2–4 Restricted	29 (5.6%)
Missing Data	236
Smoking	574 (77%)
History of autoimmune disease	50 (6.7%)
Previous chemotherapy	279 (37%)
Concurrent chemotherapy	473 (63%)
Concurrent radiotherapy	359 (48%)
Surgery for primary tumor	278 (37%)
Previous steroid use	306 (41%)
Concurrent targeted therapy	77 (10%)
Previous targeted therapy	42 (5.6%)
Previous immunotherapy use	14 (1.9%)
Previous adverse reaction to immunotherapy	6 (0.8%)
Immunotherapy type	
Atezolizumab	76 (10%)
Avelumab	3 (0.4%)
Combination Immunotherapy	77 (10%)
Durvalumab	46 (6.1%)
Ipilimumab	2 (0.3%)
Nivolumab	124 (17%)
Pembrolizumab	423 (56%)

^1^ Mean ± SD; Median [Q1, Q3]; *n* (%).

**Table 2:** irAEs Type distribution.

IRAE Type	Total *n* (%)	Grade 1 *n*	Grade 2 *n*	Grade 3 *n*	Grade 4 *n*
Endocrine IRAE	74 (9.9)	14	52	8	0
Dermatologic IRAE	68 (9.1)	27	27	13	1
Gastrointestinal IRAE	57 (7.6)	19	23	15	0
Pulmonary IRAE	35 (4.7)	4	17	13	1
Renal IRAE	26 (3.5)	8	8	9	1
Rheumatic IRAE	17 (2.3)	6	9	2	0
Neurologic IRAE	5 (0.7)	0	0	4	1
Circulatory IRAE	3 (0.4)	0	2	0	1
Systemic IRAE	16 (2.1)	8	4	4	0

**Table 3:** Multivariable Logistic Regression of Predictors per irAE System.

irAE System	Significant Predictor (Multivariable)	Adjusted OR (95% CI)	*p*-Value
Endocrine	Female Sex	1.98 (1.20–3.25)	0.007
Dermatologic	Combination Immunotherapy	2.66 (1.24–5.54)	0.013
	Prior Targeted Therapy	0.08 (0.00–0.61)	0.007
	GI Diagnosis (vs. NSCLC)	2.74 (1.12–6.34)	0.028
Gastrointestinal	Combination therapy	2.65 (1.23–5.52)	0.016
	Prior IO Adverse Reaction	7.56 (1.36–43.30)	0.023
	Age	0.87 (0.76–0.99)	0.015
	Concurrent Radiotherapy	1.82 (1.02–3.29)	0.044
Pulmonary	Atezolizumab (vs Pembrolizumab)	2.97 (1.01–8.01)	0.048
Renal	Bladder Cancer (vs. NSCLC)	4.91 (1.61–15.01)	0.004
Rheumatologic	Female Sex	4.06 (1.50–12.09)	0.007
Systemic	Bladder Cancer (vs. NSCLC)	7.11(1.35–43.71)	0.022

**Table 4:** Multivariable Logistic Regression for ORR.

Predictor	Adjusted OR (95% CI)	*p*-Value
Endocrine irAE (Yes vs. No)	2.69 (1.54–4.77)	<0.001
Dermatologic irAE (Yes vs. No)	4.07 (2.25–7.58)	<0.001
GI irAE (Yes vs. No)	2.53 (1.34–4.88)	0.005
Pulmonary irAE (Yes vs. No)	4.30 (1.89–10.38)	<0.001
Charlson Comorbidity Index	0.85 (0.79–0.91)	<0.001
Prior Steroid Use (Yes vs. No)	0.62 (0.39–0.97)	0.038
Prior Chemotherapy	0.61 (0.38–0.99)	0.047

## Data Availability

All relevant data supporting the findings of this study are included within the article. Additional data is not publicly available in order to protect patient confidentiality.

## Supplementary Materials

The following supporting information can be downloaded at: https://www.mdpi.com/article/10.3390/cancers18132167/s1, Supplementary Materials S1, Predictors; Supplementary Materials S2, OS PFS; Supplementary Materials S3, KM.
