# Supplementary material for: Organ–System Predictors of Immune–Related Adverse Events and Their Prognostic Impact in Immune Checkpoint Inhibitors–Treated Cancer Patients: A MENA Retrospective Cohort
Source: Cancers (Basel). 2026 Jul 6;18(13):2167. doi: 10.3390/cancers18132167 (PMC13359861; doi:10.3390/cancers18132167)
Supplement: Supplementary file 1 [file cancers-18-02167-s001.zip › Supplementary Materials S1-Predictors.pdf]

1. Univariate Logistic Regression: Endocrine IRAE

Predictor	Category / Comparison	Univariate OR (95% CI)	p-value
**Age (years)**	**Per 1-unit increase**	**1.00 (0.98-1.02)**	**0.732**
**BMI (kg/m2)**	**Per 1-unit increase**	**1.02 (0.97-1.07)**	**0.422**
**Charlson Comorbidity Index (per 1-point increase)**	**Per 1-unit increase**	**0.96 (0.88-1.06)**	**0.418**
Sex	***Male - Reference***	***Reference***	
	**Female vs Male**	**1.74 (1.08-2.80)**	**0.023**
Primary Diagnosis	***NSCLC - Reference***	***Reference***	
	**Bladder Cancers vs NSCLC**	**0.86 (0.35-2.15)**	**0.754**
	**GI vs NSCLC**	**0.66 (0.25-1.76)**	**0.409**
	**HPB vs NSCLC**	**0.91 (0.34-2.43)**	**0.849**
	**Melanoma vs NSCLC**	**2.04 (0.94-4.42)**	**0.071**
	**Others vs NSCLC**	**0.50 (0.22-1.17)**	**0.109**
	**RCC vs NSCLC**	**1.19 (0.50-2.83)**	**0.687**
Smoking Status	***No - Reference***	***Reference***	
	**Yes vs No**	**0.84 (0.49-1.44)**	**0.522**
Stage IV Disease	***Stage IV - Reference***	***Reference***	
	**Stage < IV vs Stage IV**	**1.17 (0.68-2.01)**	**0.582**
ECOG Performance Status	***0-1 Active - Reference***	***Reference***	
	**2-4 Restricted vs 0-1 Active**	**0.98 (0.29-3.37)**	**0.980**
Immunotherapy Type	***Pembrolizumab - Reference***	***Reference***	
	**Atezolizumab vs Pembrolizumab**	**0.79 (0.30-1.75)**	**0.575**
	**Avelumab vs Pembrolizumab**	**1.22 (0.01-12.86)**	**0.899**
	**Combo vs Pembrolizumab**	**2.11 (1.09-3.93)**	**0.027**
	**Durvalumab vs Pembrolizumab**	**0.69 (0.18-1.88)**	**0.495**
	**Ipilimumab vs Pembrolizumab**	**1.71 (0.01-21.40)**	**0.747**
	**Nivolumab vs Pembrolizumab**	**0.63 (0.27-1.28)**	**0.211**
History of Autoimmune Disease	***No - Reference***	***Reference***	

Predictor	Category / Comparison	Univariate OR (95% CI)	p-value
	Yes vs No	0.76 (0.27-2.17)	0.606
Prior Steroid Use	*No - Reference*	*Reference*	
	Yes vs No	0.64 (0.39-1.06)	0.085
Previous Chemotherapy	*No - Reference*	*Reference*	
	Yes vs No	0.53 (0.31-0.91)	0.021
Concurrent Chemotherapy	*No - Reference*	*Reference*	
	Yes vs No	0.55 (0.34-0.88)	0.014
Concurrent Radiotherapy	*No - Reference*	*Reference*	
	Yes vs No	0.68 (0.42-1.11)	0.124
Concurrent Targeted Therapy	*No - Reference*	*Reference*	
	Yes vs No	1.03 (0.48-2.24)	0.937
Prior Targeted Therapy	*No - Reference*	*Reference*	
	Yes vs No	1.85 (0.79-4.33)	0.155
Prior Adverse Reaction to Immunotherapy	*No - Reference*	*Reference*	
	Yes vs No	4.53 (0.82-25.14)	0.084

2. Univariate Logistic Regression: Renal IRAE

Predictor	Category / Comparison	Univariate OR (95% CI)	p-value
Age (years)	Per 1-unit increase	1.03 (1.00-1.07)	0.078
BMI (kg/m2)	Per 1-unit increase	1.04 (0.96-1.12)	0.354
Charlson Comorbidity Index (per 1-point increase)	Per 1-unit increase	1.05 (0.90-1.23)	0.501
Sex	*Male - Reference*	*Reference*	
	Female vs Male	0.54 (0.21-1.35)	0.187
Primary Diagnosis	*NSCLC - Reference*	*Reference*	
	Bladder Cancers vs NSCLC	5.69 (1.92-16.82)	0.002
	GI vs NSCLC	2.18 (0.55-8.64)	0.269
	HPB vs NSCLC	5.12 (1.56-16.82)	0.007
	Melanoma vs NSCLC	0.94 (0.11-7.83)	0.957
	Others vs NSCLC	0.39 (0.05-3.17)	0.376

Predictor	Category / Comparison	Univariate OR (95% CI)	p-value
	RCC vs NSCLC	1.75 (0.35-8.63)	0.494
Smoking Status	*No - Reference*	*Reference*	
	Yes vs No	1.02 (0.40-2.59)	0.962
Stage IV Disease	*Stage IV - Reference*	*Reference*	
	Stage < IV vs Stage IV	1.34 (0.54-3.32)	0.523
ECOG Performance Status	*0-1 Active - Reference*	*Reference*	
	2-4 Restricted vs 0-1 Active	2.93 (0.62-13.74)	0.174
Immunotherapy Type	*Pembrolizumab - Reference*	*Reference*	
	Atezolizumab vs Pembrolizumab	2.53 (0.83-6.83)	0.098
	Avelumab vs Pembrolizumab	4.70 (0.03-52.51)	0.400
	Combo vs Pembrolizumab	1.55 (0.39-4.73)	0.497
	Durvalumab vs Pembrolizumab	0.35 (0.00-2.77)	0.400
	Ipilimumab vs Pembrolizumab	6.58 (0.05-86.73)	0.328
	Nivolumab vs Pembrolizumab	1.81 (0.64-4.63)	0.248
History of Autoimmune Disease	*No - Reference*	*Reference*	
	Yes vs No	1.88 (0.55-6.50)	0.317
Prior Steroid Use	*No - Reference*	*Reference*	
	Yes vs No	0.90 (0.40-2.02)	0.805
Previous Chemotherapy	*No - Reference*	*Reference*	
	Yes vs No	0.89 (0.39-2.03)	0.781
Concurrent Chemotherapy	*No - Reference*	*Reference*	
	Yes vs No	0.58 (0.26-1.26)	0.168
Concurrent Radiotherapy	*No - Reference*	*Reference*	
	Yes vs No	0.39 (0.16-0.94)	0.036
Concurrent Targeted Therapy	*No - Reference*	*Reference*	
	Yes vs No	2.16 (0.79-5.90)	0.133
Prior Targeted Therapy	*No - Reference*	*Reference*	
	Yes vs No	1.43 (0.33-6.25)	0.637
Prior Adverse Reaction to Immunotherapy	*No - Reference*	*Reference*	

Predictor	Category / Comparison	Univariate OR (95% CI)	p-value
	**Yes vs No**	**5.76 (0.65-51.15)**	**0.116**

3. Univariate Logistic Regression: Dermatologic IRAE

Predictor	Category / Comparison	Univariate OR (95% CI)	p-value
**Age (years)**	**Per 1-unit increase**	**1.01 (0.99-1.03)**	**0.453**
**BMI (kg/m2)**	**Per 1-unit increase**	**0.99 (0.94-1.05)**	**0.819**
**Charlson Comorbidity Index (per 1-point increase)**	**Per 1-unit increase**	**1.00 (0.91-1.11)**	**0.952**
Sex	***Male - Reference***	***Reference***	
	**Female vs Male**	**0.80 (0.47-1.36)**	**0.412**
Primary Diagnosis	***NSCLC - Reference***	***Reference***	
	**Bladder Cancers vs NSCLC**	**1.27 (0.50-3.23)**	**0.617**
	**GI vs NSCLC**	**1.90 (0.85-4.28)**	**0.120**
	**HPB vs NSCLC**	**1.36 (0.49-3.72)**	**0.554**
	**Melanoma vs NSCLC**	**1.36 (0.49-3.72)**	**0.554**
	**Others vs NSCLC**	**1.47 (0.73-2.98)**	**0.280**
	**RCC vs NSCLC**	**2.08 (0.89-4.88)**	**0.092**
Smoking Status	***No - Reference***	***Reference***	
	**Yes vs No**	**0.97 (0.55-1.72)**	**0.921**
Stage IV Disease	***Stage IV - Reference***	***Reference***	
	**Stage < IV vs Stage IV**	**1.10 (0.63-1.95)**	**0.731**
ECOG Performance Status	***0-1 Active - Reference***	***Reference***	
	**2-4 Restricted vs 0-1 Active**	**0.71 (0.16-3.08)**	**0.646**
Immunotherapy Type	***Pembrolizumab - Reference***	***Reference***	
	**Atezolizumab vs Pembrolizumab**	**0.77 (0.27-1.81)**	**0.571**
	**Avelumab vs Pembrolizumab**	**1.43 (0.01-15.15)**	**0.822**
	**Combo vs Pembrolizumab**	**2.48 (1.27-4.67)**	**0.009**
	**Durvalumab vs Pembrolizumab**	**1.33 (0.46-3.19)**	**0.570**

Predictor	Category / Comparison	Univariate OR (95% CI)	p-value
	Ipilimumab vs Pembrolizumab	2.00 (0.01-25.20)	0.680
	Nivolumab vs Pembrolizumab	0.73 (0.32-1.51)	0.412
History of Autoimmune Disease	*No - Reference*	*Reference*	
	Yes vs No	1.93 (0.87-4.29)	0.107
Prior Steroid Use	*No - Reference*	*Reference*	
	Yes vs No	0.88 (0.53-1.45)	0.618
Previous Chemotherapy	*No - Reference*	*Reference*	
	Yes vs No	0.74 (0.44-1.25)	0.256
Concurrent Chemotherapy	*No - Reference*	*Reference*	
	Yes vs No	0.50 (0.31-0.82)	0.006
Concurrent Radiotherapy	*No - Reference*	*Reference*	
	Yes vs No	0.89 (0.54-1.45)	0.628
Concurrent Targeted Therapy	*No - Reference*	*Reference*	
	Yes vs No	1.50 (0.73-3.06)	0.266
Prior Targeted Therapy	*No - Reference*	*Reference*	
	Yes vs No	0.11 (0.00-0.75)	0.017
Prior Adverse Reaction to Immunotherapy	*No - Reference*	*Reference*	
	Yes vs No	0.73 (0.01-6.24)	0.820

4. Univariate Logistic Regression: GI IRAE

Predictor	Category / Comparison	Univariate OR (95% CI)	p-value
Age (years)	Per 1-unit increase	0.97 (0.96-0.99)	0.008
BMI (kg/m2)	Per 1-unit increase	0.98 (0.93-1.04)	0.491
Charlson Comorbidity Index (per 1-point increase)	Per 1-unit increase	0.95 (0.86-1.06)	0.382
Sex	*Male - Reference*	*Reference*	
	Female vs Male	1.22 (0.70-2.11)	0.483
Primary Diagnosis	*NSCLC - Reference*	*Reference*	
	Bladder Cancers vs NSCLC	1.22 (0.42-3.00)	0.696

Predictor	Category / Comparison	Univariate OR (95% CI)	p-value
	GI vs NSCLC	1.16 (0.40-2.85)	0.768
	HPB vs NSCLC	0.13 (0.00-0.96)	0.044
	Melanoma vs NSCLC	2.26 (0.88-5.23)	0.087
	Others vs NSCLC	1.24 (0.56-2.58)	0.586
	RCC vs NSCLC	2.35 (0.96-5.27)	0.060
Smoking Status	*No - Reference*	*Reference*	
	Yes vs No	1.07 (0.56-2.03)	0.844
Stage IV Disease	*Stage IV - Reference*	*Reference*	
	Stage < IV vs Stage IV	0.84 (0.43-1.63)	0.600
ECOG Performance Status	*0-1 Active - Reference*	*Reference*	
	2-4 Restricted vs 0-1 Active	2.39 (0.86-6.61)	0.094
Immunotherapy Type	*Pembrolizumab - Reference*	*Reference*	
	Atezolizumab vs Pembrolizumab	1.19 (0.45-2.72)	0.705
	Avelumab vs Pembrolizumab	1.84 (0.01-19.65)	0.709
	Combo vs Pembrolizumab	3.46 (1.76-6.61)	<0.001
	Durvalumab vs Pembrolizumab	0.14 (0.00-1.01)	0.052
	Ipilimumab vs Pembrolizumab	2.58 (0.02-32.65)	0.584
	Nivolumab vs Pembrolizumab	0.71 (0.27-1.59)	0.422
History of Autoimmune Disease	*No - Reference*	*Reference*	
	Yes vs No	1.36 (0.52-3.57)	0.534
Prior Steroid Use	*No - Reference*	*Reference*	
	Yes vs No	0.81 (0.46-1.41)	0.459
Previous Chemotherapy	*No - Reference*	*Reference*	
	Yes vs No	0.57 (0.31-1.04)	0.066
Concurrent Chemotherapy	*No - Reference*	*Reference*	
	Yes vs No	0.52 (0.30-0.89)	0.017
Concurrent Radiotherapy	*No - Reference*	*Reference*	
	Yes vs No	1.60 (0.93-2.76)	0.088
Concurrent Targeted Therapy	*No - Reference*	*Reference*	

Predictor	Category / Comparison	Univariate OR (95% CI)	p-value
	Yes vs No	1.01 (0.42-2.44)	0.981
Prior Targeted Therapy	*No - Reference*	*Reference*	
	Yes vs No	0.91 (0.27-3.06)	0.885
Prior Adverse Reaction to Immunotherapy	*No - Reference*	*Reference*	
	Yes vs No	12.55 (2.47-63.63)	0.002

5. Univariate Logistic Regression: Pulmonary IRAE

Predictor	Category / Comparison	Univariate OR (95% CI)	p-value
Age (years)	Per 1-unit increase	1.00 (0.98-1.03)	0.764
BMI (kg/m2)	Per 1-unit increase	1.01 (0.94-1.08)	0.878
Charlson Comorbidity Index (per 1-point increase)	Per 1-unit increase	0.99 (0.86-1.13)	0.843
Sex	*Male - Reference*	*Reference*	
	Female vs Male	1.57 (0.79-3.11)	0.195
Primary Diagnosis	*NSCLC - Reference*	*Reference*	
	Bladder Cancers vs NSCLC	0.48 (0.10-1.53)	0.239
	GI vs NSCLC	0.27 (0.03-1.07)	0.066
	HPB vs NSCLC	0.12 (0.00-0.88)	0.032
	Melanoma vs NSCLC	0.88 (0.23-2.50)	0.833
	Others vs NSCLC	0.25 (0.05-0.79)	0.015
	RCC vs NSCLC	0.56 (0.11-1.79)	0.362
Smoking Status	*No - Reference*	*Reference*	
	Yes vs No	1.04 (0.46-2.32)	0.931
Stage IV Disease	*Stage IV - Reference*	*Reference*	
	Stage < IV vs Stage IV	1.63 (0.79-3.34)	0.185
ECOG Performance Status	*0-1 Active - Reference*	*Reference*	
	2-4 Restricted vs 0-1 Active	1.31 (0.30-5.81)	0.722
Immunotherapy Type	*Pembrolizumab - Reference*	*Reference*	
	Atezolizumab vs Pembrolizumab	2.28 (0.83-5.61)	0.106

Predictor	Category / Comparison	Univariate OR (95% CI)	p-value
	Avelumab vs Pembrolizumab	3.53 (0.03-38.65)	0.478
	Combo vs Pembrolizumab	1.51 (0.46-4.10)	0.466
	Durvalumab vs Pembrolizumab	3.27 (1.08-8.59)	0.037
	Ipilimumab vs Pembrolizumab	4.94 (0.04-63.99)	0.392
	Nivolumab vs Pembrolizumab	0.92 (0.28-2.47)	0.880
History of Autoimmune Disease	*No - Reference*	*Reference*	
	Yes vs No	0.84 (0.20-3.62)	0.819
Prior Steroid Use	*No - Reference*	*Reference*	
	Yes vs No	1.77 (0.90-3.50)	0.100
Previous Chemotherapy	*No - Reference*	*Reference*	
	Yes vs No	1.28 (0.64-2.55)	0.479
Concurrent Chemotherapy	*No - Reference*	*Reference*	
	Yes vs No	2.04 (0.92-4.56)	0.081
Concurrent Radiotherapy	*No - Reference*	*Reference*	
	Yes vs No	1.90 (0.94-3.84)	0.072
Concurrent Targeted Therapy	*No - Reference*	*Reference*	
	Yes vs No	0.52 (0.12-2.20)	0.373
Prior Targeted Therapy	*No - Reference*	*Reference*	
	Yes vs No	0.48 (0.06-3.63)	0.480
Prior Adverse Reaction to Immunotherapy	*No - Reference*	*Reference*	
	Yes vs No	4.18 (0.48-36.79)	0.197

6. Univariate Logistic Regression: Systemic/Other IRAE

Predictor	Category / Comparison	Univariate OR (95% CI)	p-value
Age (years)	Per 1-unit increase	0.96 (0.94-0.99)	0.011
BMI (kg/m2)	Per 1-unit increase	1.00 (0.91-1.09)	0.975
Charlson Comorbidity Index (per 1-point increase)	Per 1-unit increase	0.82 (0.69-0.98)	0.028

Predictor	Category / Comparison	Univariate OR (95% CI)	p-value
Sex	*Male - Reference*	*Reference*	
	Female vs Male	1.38 (0.57-3.32)	0.472
Primary Diagnosis	*NSCLC - Reference*	*Reference*	
	Bladder Cancers vs NSCLC	7.52 (1.43-45.91)	0.019
	GI vs NSCLC	2.98 (0.27-22.78)	0.324
	HPB vs NSCLC	1.30 (0.01-16.31)	0.869
	Melanoma vs NSCLC	22.62 (5.82-124.04)	<0.001
	Others vs NSCLC	2.76 (0.42-18.05)	0.269
	RCC vs NSCLC	17.27 (4.28-96.06)	<0.001
Smoking Status	*No - Reference*	*Reference*	
	Yes vs No	0.49 (0.20-1.19)	0.116
Stage IV Disease	*Stage IV - Reference*	*Reference*	
	Stage < IV vs Stage IV	1.66 (0.65-4.23)	0.287
ECOG Performance Status	*0-1 Active - Reference*	*Reference*	
	2-4 Restricted vs 0-1 Active	2.13 (0.26-17.66)	0.482
Immunotherapy Type	*Pembrolizumab - Reference*	*Reference*	
	Atezolizumab vs Pembrolizumab	0.29 (0.00-2.29)	0.297
	Avelumab vs Pembrolizumab	6.23 (0.05-71.40)	0.336
	Combo vs Pembrolizumab	4.64 (1.67-12.49)	0.004
	Durvalumab vs Pembrolizumab	0.47 (0.00-3.80)	0.561
	Ipilimumab vs Pembrolizumab	8.73 (0.06-117.52)	0.276
	Nivolumab vs Pembrolizumab	2.01 (0.64-5.71)	0.218
History of Autoimmune Disease	*No - Reference*	*Reference*	
	Yes vs No	2.42 (0.69-8.52)	0.168
Prior Steroid Use	*No - Reference*	*Reference*	
	Yes vs No	0.33 (0.11-1.00)	0.050
Previous Chemotherapy	*No - Reference*	*Reference*	
	Yes vs No	0.52 (0.19-1.43)	0.205
Concurrent Chemotherapy	*No - Reference*	*Reference*	

Predictor	Category / Comparison	Univariate OR (95% CI)	p-value
	Yes vs No	0.23 (0.09-0.59)	0.002
Concurrent Radiotherapy	*No - Reference*	*Reference*	
	Yes vs No	0.33 (0.12-0.92)	0.033
Concurrent Targeted Therapy	*No - Reference*	*Reference*	
	Yes vs No	1.48 (0.43-5.13)	0.539
Prior Targeted Therapy	*No - Reference*	*Reference*	
	Yes vs No	0.84 (0.11-6.42)	0.867
Prior Adverse Reaction to Immunotherapy	*No - Reference*	*Reference*	
	Yes vs No	2.59 (0.02-23.15)	0.571

7. Univariate Logistic Regression: Rheumatologic IRAE

Predictor	Category / Comparison	Univariate OR (95% CI)	p-value
Age (years)	Per 1-unit increase	1.03 (0.98-1.07)	0.232
BMI (kg/m2)	Per 1-unit increase	0.95 (0.86-1.06)	0.398
Charlson Comorbidity Index (per 1-point increase)	Per 1-unit increase	1.12 (0.93-1.36)	0.243
Sex	*Male - Reference*	*Reference*	
	Female vs Male	3.44 (1.26-9.40)	0.016
Primary Diagnosis	*NSCLC - Reference*	*Reference*	
	Bladder Cancers vs NSCLC	0.22 (0.00-1.70)	0.180
	GI vs NSCLC	0.63 (0.07-2.71)	0.579
	HPB vs NSCLC	0.28 (0.00-2.17)	0.278
	Melanoma vs NSCLC	0.84 (0.09-3.66)	0.844
	Others vs NSCLC	0.82 (0.21-2.54)	0.752
	RCC vs NSCLC	0.78 (0.08-3.38)	0.773
Smoking Status	*No - Reference*	*Reference*	
	Yes vs No	1.44 (0.41-5.08)	0.567
Stage IV Disease	*Stage IV - Reference*	*Reference*	
	Stage < IV vs Stage IV	0.64 (0.18-2.27)	0.493
ECOG Performance Status	*0-1 Active - Reference*	*Reference*	

Predictor	Category / Comparison	Univariate OR (95% CI)	p-value
	**2-4 Restricted vs 0-1 Active**	**1.89 (0.23-15.44)**	**0.553**
Immunotherapy Type	***Pembrolizumab - Reference***	***Reference***	
	**Atezolizumab vs Pembrolizumab**	**0.78 (0.08-3.41)**	**0.774**
	**Avelumab vs Pembrolizumab**	**5.63 (0.04-63.79)**	**0.358**
	**Combo vs Pembrolizumab**	**2.44 (0.71-7.20)**	**0.146**
	**Durvalumab vs Pembrolizumab**	**0.42 (0.00-3.38)**	**0.501**
	**Ipilimumab vs Pembrolizumab**	**7.88 (0.06-105.14)**	**0.294**
	**Nivolumab vs Pembrolizumab**	**0.80 (0.15-2.82)**	**0.754**
History of Autoimmune Disease	***No - Reference***	***Reference***	
	**Yes vs No**	**1.95 (0.43-8.76)**	**0.386**
Prior Steroid Use	***No - Reference***	***Reference***	
	**Yes vs No**	**0.60 (0.21-1.71)**	**0.337**
Previous Chemotherapy	***No - Reference***	***Reference***	
	**Yes vs No**	**0.92 (0.34-2.51)**	**0.866**
Concurrent Chemotherapy	***No - Reference***	***Reference***	
	**Yes vs No**	**0.40 (0.15-1.07)**	**0.069**
Concurrent Radiotherapy	***No - Reference***	***Reference***	
	**Yes vs No**	**0.59 (0.22-1.61)**	**0.304**
Concurrent Targeted Therapy	***No - Reference***	***Reference***	
	**Yes vs No**	**0.55 (0.07-4.19)**	**0.563**
Prior Targeted Therapy	***No - Reference***	***Reference***	
	**Yes vs No**	**1.08 (0.14-8.37)**	**0.939**
Prior Adverse Reaction to Immunotherapy	***No - Reference***	***Reference***	
	**Yes vs No**	**3.20 (0.02-28.92)**	**0.502**
