# Supplementary material for: Organ–System Predictors of Immune–Related Adverse Events and Their Prognostic Impact in Immune Checkpoint Inhibitors–Treated Cancer Patients: A MENA Retrospective Cohort
Source: Cancers (Basel). 2026 Jul 6;18(13):2167. doi: 10.3390/cancers18132167 (PMC13359861; doi:10.3390/cancers18132167)
Supplement: Supplementary file 1 [file cancers-18-02167-s001.zip › Supplementary Materials S2-OS PFS.pdf]

1.1. Univariate Cox Regression: Overall Survival (OS)

N=731 patients | Events=209 deaths (28.6%)

Variable	Category	HR_CI	p_value	n_events
Endocrine IRAE	*No (reference)*	*Reference*		
	Yes	0.74 (0.46–1.19)	0.213	209
Dermatologic IRAE	*No (reference)*	*Reference*		
	Yes	0.62 (0.37–1.03)	0.066	209
GI IRAE	*No (reference)*	*Reference*		
	Yes	0.98 (0.61–1.57)	0.927	209
Pulmonary IRAE	*No (reference)*	*Reference*		
	Yes	0.98 (0.57–1.69)	0.938	209
Age (years)	Per 1-unit increase	1.00 (0.99–1.01)	0.577	209
BMI (kg/m2)	Per 1-unit increase	0.98 (0.96–1.01)	0.259	209
Charlson Comorbidity Index	Per 1-unit increase	1.14 (1.09–1.21)	**<0.001**	208
Sex	*Male (reference)*	*Reference*		
	Female	0.90 (0.68–1.19)	0.445	209
Primary Diagnosis	*NSCLC (reference)*	*Reference*		
	Bladder Cancers	0.82 (0.48–1.41)	0.468	209
	Gastrointestinal	1.29 (0.82–2.03)	0.269	209
	Hepatobiliary	0.77 (0.38–1.59)	0.486	209
	Melanoma	0.95 (0.52–1.74)	0.879	209
	Others	1.15 (0.78–1.68)	0.481	209
	Renal cell carcinoma	0.71 (0.39–1.30)	0.268	209
Smoking Status	*No (reference)*	*Reference*		
	Yes	0.97 (0.71–1.32)	0.828	209
ECOG Performance Status	*0-1 Active (reference)*	*Reference*		
	2-4 Restricted	2.46 (1.50–4.04)	**<0.001**	151
Immunotherapy Type	*Pembrolizumab (reference)*	*Reference*		
	Atezolizumab	0.92 (0.57–1.50)	0.743	209
	Avelumab	-	0.993	209
	Combination Immunotherapy	1.52 (1.01–2.29)	**0.043**	209
	Durvalumab	0.88 (0.47–1.63)	0.682	209
	Ipilimumab	-	0.997	209
	Nivolumab	1.24 (0.85–1.81)	0.270	209
History of Autoimmune Disease	*No (reference)*	*Reference*		
	Yes	1.19 (0.74–1.94)	0.472	209
Prior Steroid Use	*No (reference)*	*Reference*		
	Yes	1.41 (1.08–1.86)	**0.013**	209
Previous Chemotherapy	*No (reference)*	*Reference*		

Variable	Category	HR_CI	p_value	n_events
	Yes	0.97 (0.73–1.29)	0.848	209
Concurrent Chemotherapy	*No (reference)*	*Reference*		
	Yes	1.22 (0.92–1.63)	0.173	209
Concurrent Radiotherapy	*No (reference)*	*Reference*		
	Yes	1.05 (0.80–1.38)	0.712	209
Concurrent Targeted Therapy	*No (reference)*	*Reference*		
	Yes	1.08 (0.70–1.65)	0.734	209
Prior Targeted Therapy	*No (reference)*	*Reference*		
	Yes	1.32 (0.75–2.31)	0.336	209
Prior Adverse Reaction to Immunotherapy	*No (reference)*	*Reference*		
	Yes	0.49 (0.07–3.50)	0.477	209
Surgery for Primary Tumor	*No (reference)*	*Reference*		
	Yes	0.70 (0.53–0.94)	**0.018**	209
Previous Use of Immunotherapy	*No (reference)*	*Reference*		
	Yes	2.53 (1.12–5.72)	**0.025**	209

1.2. Univariate Cox Regression: Progression-Free Survival (PFS)

N=731 patients | Events=180 progressions/deaths (24.6%)

Variable	Category	HR_CI	p_value	n_events
Endocrine IRAE	*No (reference)*	*Reference*		
	Yes	1.19 (0.77–1.83)	0.440	173
Dermatologic IRAE	*No (reference)*	*Reference*		
	Yes	1.33 (0.88–2.01)	0.170	173
GI IRAE	*No (reference)*	*Reference*		
	Yes	0.60 (0.32–1.10)	0.097	173
Pulmonary IRAE	*No (reference)*	*Reference*		
	Yes	1.42 (0.85–2.38)	0.184	173
Age (years)	Per 1-unit increase	1.01 (1.00–1.02)	0.148	173
BMI (kg/m2)	Per 1-unit increase	0.99 (0.96–1.02)	0.483	169
Charlson Comorbidity Index	Per 1-unit increase	0.99 (0.93–1.04)	0.650	171
Sex	*Male (reference)*	*Reference*		
	Female	1.05 (0.78–1.43)	0.742	173
Primary Diagnosis	*NSCLC (reference)*	*Reference*		
	Bladder Cancers	0.25 (0.10–0.62)	**0.003**	173
	Gastrointestinal	0.85 (0.48–1.48)	0.557	173
	Hepatobiliary	1.01 (0.52–1.93)	0.986	173
	Melanoma	0.22 (0.07–0.69)	**0.010**	173
	Others	1.01 (0.68–1.51)	0.950	173

Variable	Category	HR_CI	p_value	n_events
	Renal cell carcinoma	0.26 (0.11–0.65)	**0.004**	173
Smoking Status	*No (reference)*	*Reference*		
	Yes	1.51 (1.02–2.23)	0.059	173
ECOG Performance Status	*0-1 Active (reference)*	*Reference*		
	2-4 Restricted	0.71 (0.29–1.74)	0.456	133
Immunotherapy Type	*Pembrolizumab (reference)*	*Reference*		
	Atezolizumab	1.56 (1.02–2.39)	0.060	173
	Avelumab	-	0.993	173
	Combo	0.97 (0.57–1.64)	0.900	173
	Durvalumab	1.17 (0.65–2.08)	0.601	173
	Ipilimumab	-	0.997	173
	Nivolumab	0.81 (0.49–1.33)	0.402	173
History of Autoimmune Disease	*No (reference)*	*Reference*		
	Yes	1.00 (0.57–1.76)	0.992	173
Prior Steroid Use	*No (reference)*	*Reference*		
	Yes	0.98 (0.72–1.34)	0.916	173
Previous Chemotherapy	*No (reference)*	*Reference*		
	Yes	0.88 (0.64–1.21)	0.433	173
Concurrent Chemotherapy	*No (reference)*	*Reference*		
	Yes	1.98 (1.40–2.80)	**<0.001**	173
Concurrent Radiotherapy	*No (reference)*	*Reference*		
	Yes	1.03 (0.77–1.39)	0.827	173
Concurrent Targeted Therapy	*No (reference)*	*Reference*		
	Yes	0.68 (0.39–1.18)	0.168	173
Prior Targeted Therapy	*No (reference)*	*Reference*		
	Yes	0.79 (0.35–1.77)	0.562	173
Prior Adverse Reaction to Immunotherapy	*No (reference)*	*Reference*		
	Yes	-	0.992	173
Surgery for Primary Tumor	*No (reference)*	*Reference*		
	Yes	0.51 (0.36–0.71)	**<0.001**	172
Previous Use of Immunotherapy	*No (reference)*	*Reference*		
	Yes	1.48 (0.36–5.98)	0.585	173

1.1. Multivariate Cox Regression: Overall Survival (OS)

N=498 patients | Events=151 deaths

Variable	Category	HR_CI	p_value	n_events
Endocrine IRAE	*No (reference)*	*Reference*		
	Yes	0.60 (0.32–1.12)	0.107	151
Dermatologic IRAE	*No (reference)*	*Reference*		
	Yes	0.43 (0.22–0.85)	0.055	151
GI IRAE	*No (reference)*	*Reference*		
	Yes	0.71 (0.39–1.28)	0.249	151
Pulmonary IRAE	*No (reference)*	*Reference*		
	Yes	0.71 (0.37–1.36)	0.304	151
Sex	*Male (reference)*	*Reference*		
	Female	0.93 (0.65–1.34)	0.712	151
BMI (kg/m2)	Per 1-unit increase	0.97 (0.93–1.00)	0.076	151
Charlson Comorbidity Index	Per 1-unit increase	1.19 (1.10–1.28)	**<0.001**	151
ECOG Performance Status	*0-1 Active (reference)*	*Reference*		
	2-4 Restricted	1.88 (1.09–3.25)	**0.024**	151
Immunotherapy Type	*Pembrolizumab (reference)*	*Reference*		
	Atezolizumab	1.35 (0.78–2.35)	0.279	151
	Avelumab	-	-	151
	Combination Immunotherapy	1.78 (1.08–2.92)	**0.023**	151
	Durvalumab	1.03 (0.52–2.03)	0.927	151
	Ipilimumab	-	0.993	151
	Nivolumab	1.26 (0.75–2.13)	0.383	151
Prior Steroid Use	*No (reference)*	*Reference*		
	Yes	1.31 (0.92–1.87)	0.134	151
Surgery for Primary Tumor	*No (reference)*	*Reference*		
	Yes	0.77 (0.53–1.12)	0.175	151
Previous Use of Immunotherapy	*No (reference)*	*Reference*		
	Yes	2.42 (0.84–6.95)	0.101	151

1.2. Multivariate Cox Regression: Progression-Free Survival (PFS)

N=494 patients | Events=131 progressions

Variable	Category	HR_CI	p_value	n_events
Endocrine IRAE	*No (reference)*	*Reference*		
	Yes	1.21 (0.72–2.06)	0.472	131
Dermatologic IRAE	*No (reference)*	*Reference*		
	Yes	1.40 (0.82–2.39)	0.218	131
Gastrointestinal IRAE	*No (reference)*	*Reference*		
	Yes	0.49 (0.23–1.03)	0.062	131
Pulmonary IRAE	*No (reference)*	*Reference*		
	Yes	1.25 (0.67–2.32)	0.484	131
Sex	*Male (reference)*	*Reference*		
	Female	1.16 (0.80–1.69)	0.433	131
BMI (kg/m2)	Per 1-unit increase	1.00 (0.96–1.04)	0.891	131
Charlson Comorbidity Index	Per 1-unit increase	0.95 (0.88–1.03)	0.202	131
ECOG Performance Status	*0-1 Active (reference)*	*Reference*		
	2-4 Restricted	1.08 (0.41–2.85)	0.871	131
Primary Diagnosis	*NSCLC (reference)*	*Reference*		
	Bladder Cancers	0.49 (0.16–1.45)	0.195	131
	Gastrointestinal	1.14 (0.53–2.47)	0.739	131
	Hepatobiliary	2.04 (0.67–6.20)	0.206	131
	Melanoma	0.40 (0.08–1.93)	0.257	131
	Others	1.43 (0.84–2.43)	0.193	131
	Renal cell carcinoma	0.70 (0.26–1.93)	0.497	131
Smoking Status	*No (reference)*	*Reference*		
	Yes	1.51 (0.90–2.53)	0.118	131
Immunotherapy Type	*Pembrolizumab (reference)*	*Reference*		
	Atezolizumab	1.26 (0.72–2.20)	0.411	131
	Avelumab	-	-	131
	Combination immunotherapy	1.81 (0.95–3.46)	0.070	131
	Durvalumab	0.64 (0.32–1.28)	0.202	131
	Ipilimumab	-	0.996	131
	Nivolumab	0.91 (0.45–1.84)	0.801	131
Concurrent Chemotherapy	*No (reference)*	*Reference*		
	Yes	2.30 (1.33–3.98)	**0.003**	131
Surgery for Primary Tumor	*No (reference)*	*Reference*		
	Yes	0.96 (0.62–1.49)	0.852	131
