# Supplementary material for: Organ–System Predictors of Immune–Related Adverse Events and Their Prognostic Impact in Immune Checkpoint Inhibitors–Treated Cancer Patients: A MENA Retrospective Cohort
Source: Cancers (Basel). 2026 Jul 6;18(13):2167. doi: 10.3390/cancers18132167 (PMC13359861; doi:10.3390/cancers18132167)
Supplement: Supplementary file 1 [file cancers-18-02167-s001.zip › Supplementary Materials S3-KM.pdf]

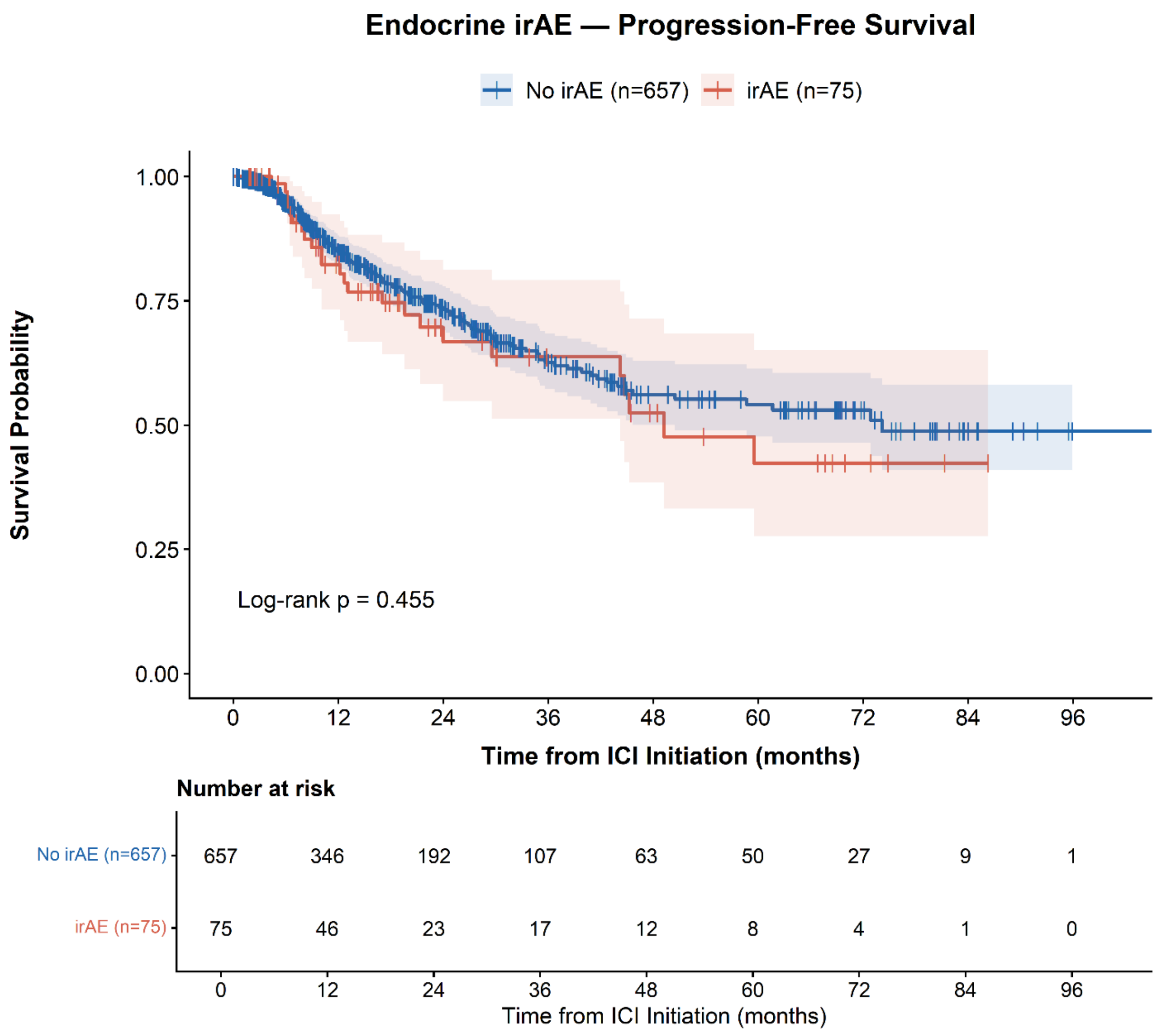
Endocrine irAE — Progression-Free Survival
No irAE (n=657)
irAE (n=75)
Survival Probability
1.00
0.75
0.50
0.25
0.00
Log-rank p = 0.455
0
12
24
36
48
60
72
84
96
Time from ICI Initiation (months)
Number at risk
No irAE (n=657) 657 346 192 107 63 50 27 9 1
irAE (n=75) 75 46 23 17 12 8 4 1 0
Time from ICI Initiation (months)

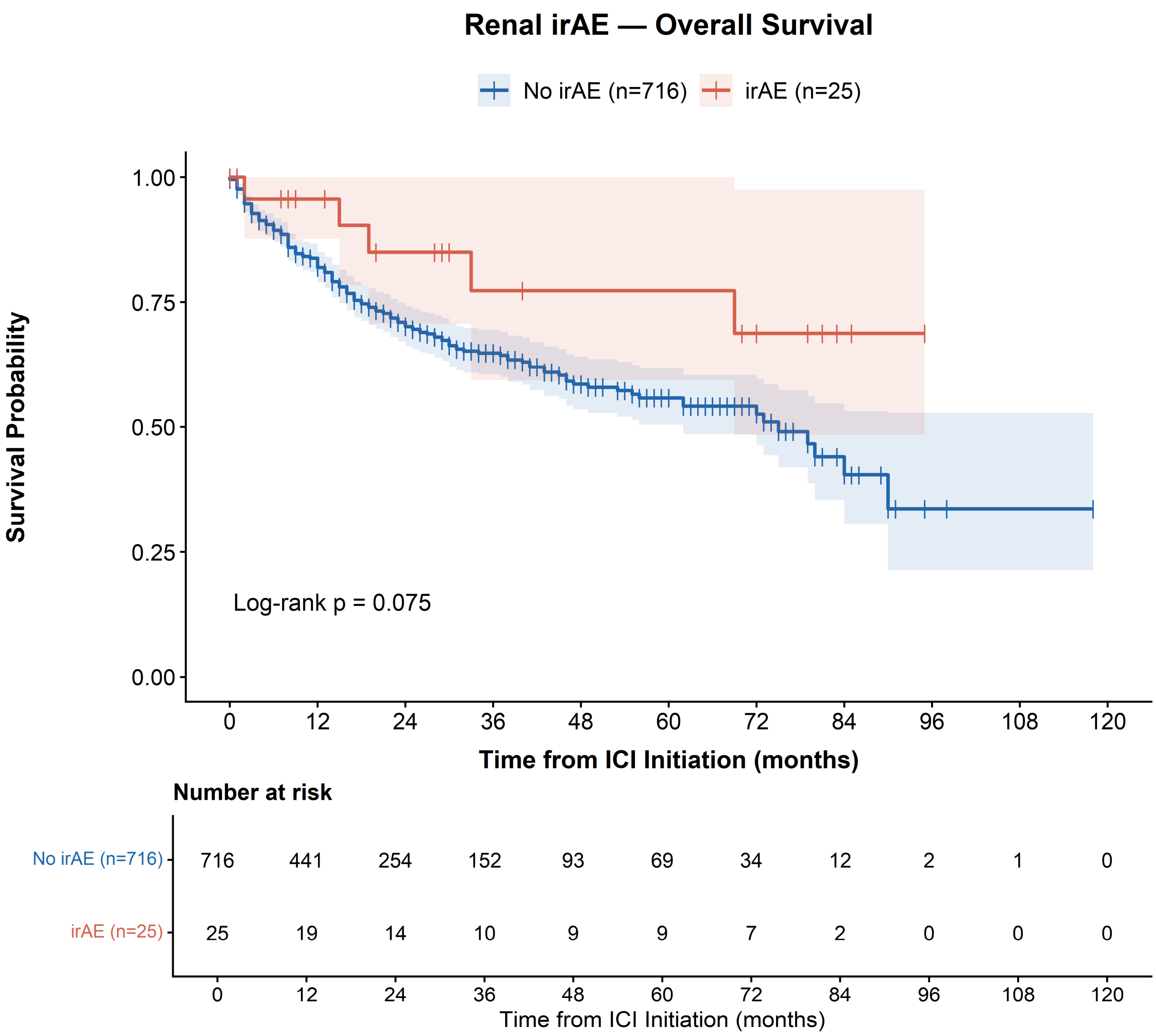
Renal irAE — Overall Survival
No irAE (n=716)
irAE (n=25)
Survival Probability
1.00
0.75
0.50
0.25
0.00
Log-rank p = 0.075
0
12
24
36
48
60
72
84
96
108
120
Time from ICI Initiation (months)
Number at risk
No irAE (n=716) 716 441 254 152 93 69 34 12 2 1 0
irAE (n=25) 25 19 14 10 9 9 7 2 0 0 0
0
12
24
36
48
60
72
84
96
108
120
Time from ICI Initiation (months)

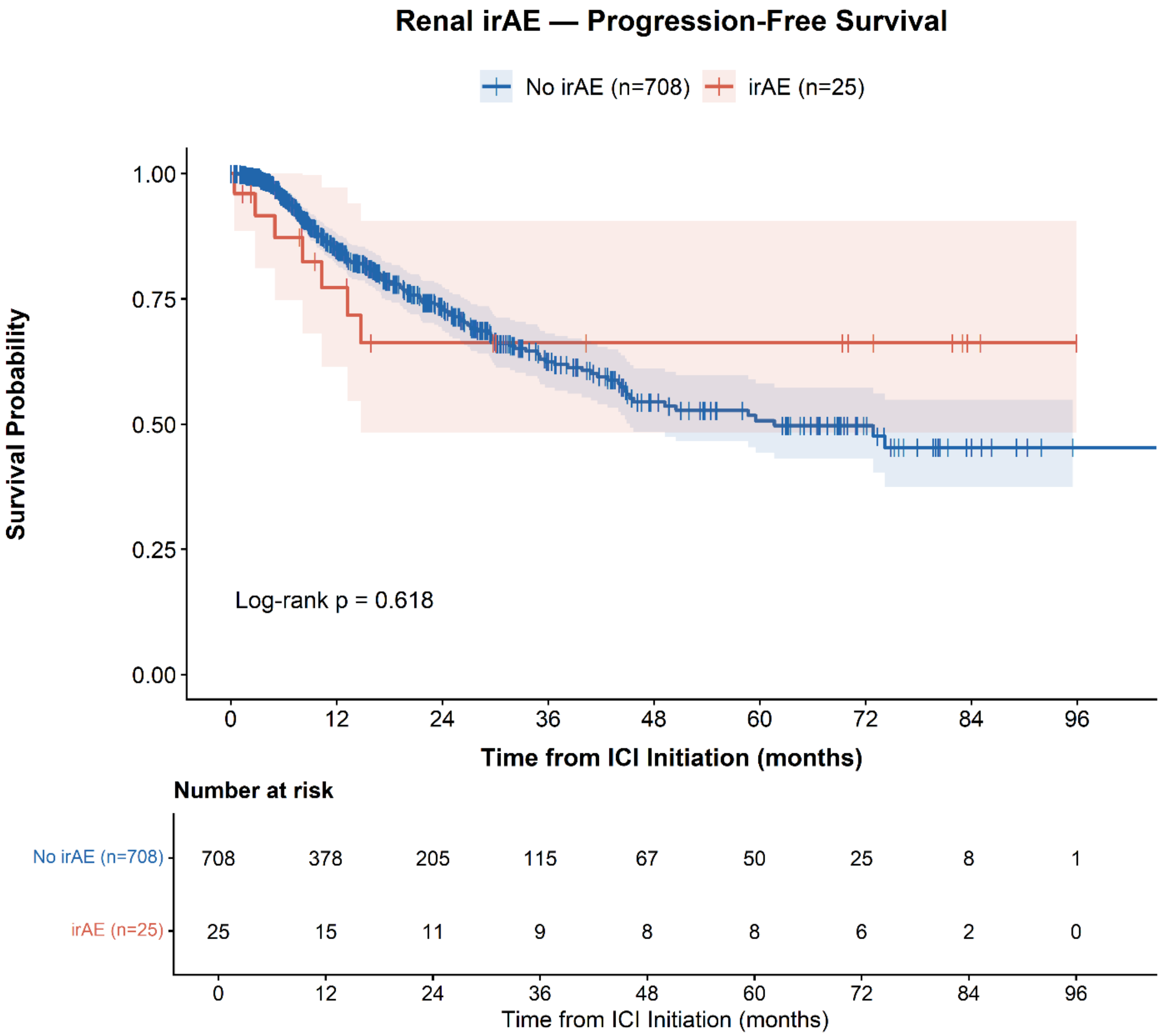

Renal irAE — Progression-Free Survival
No irAE (n=708)
irAE (n=25)
Survival Probability
1.00
0.75
0.50
0.25
0.00
Log-rank p = 0.618
0
12
24
36
48
60
72
84
96
Time from ICI Initiation (months)
Number at risk
No irAE (n=708) 708 378 205 115 67 50 25 8 1
irAE (n=25) 25 15 11 9 8 8 6 2 0
0
12
24
36
48
60
72
84
96
Time from ICI Initiation (months)

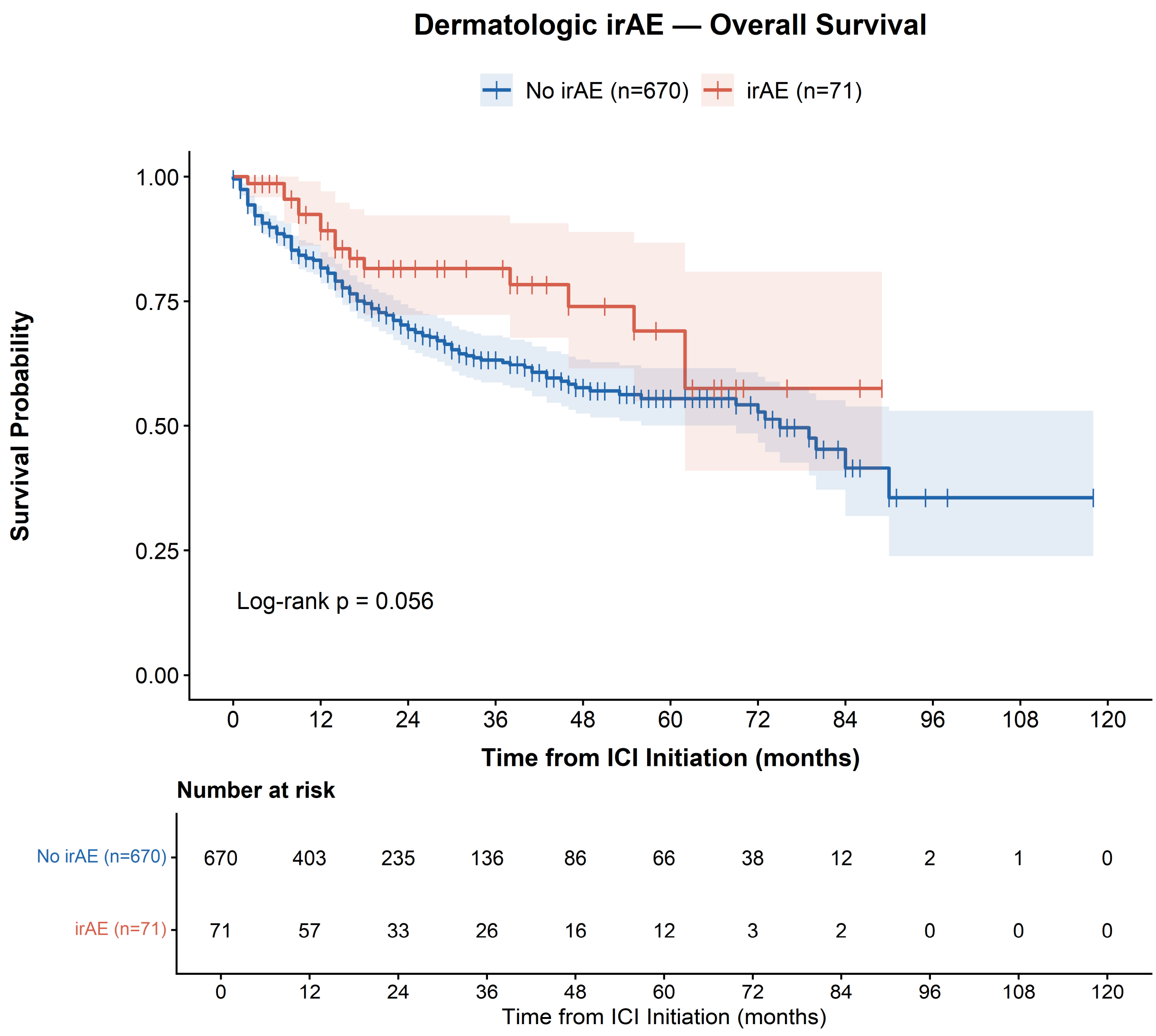
Dermatologic irAE — Overall Survival
No irAE (n=670)
irAE (n=71)
Survival Probability
1.00
0.75
0.50
0.25
0.00
Log-rank p = 0.056
0
12
24
36
48
60
72
84
96
108
120
Time from ICI Initiation (months)
Number at risk
No irAE (n=670) 670 403 235 136 86 66 38 12 2 1 0
irAE (n=71) 71 57 33 26 16 12 3 2 0 0 0
0
12
24
36
48
60
72
84
96
108
120
Time from ICI Initiation (months)

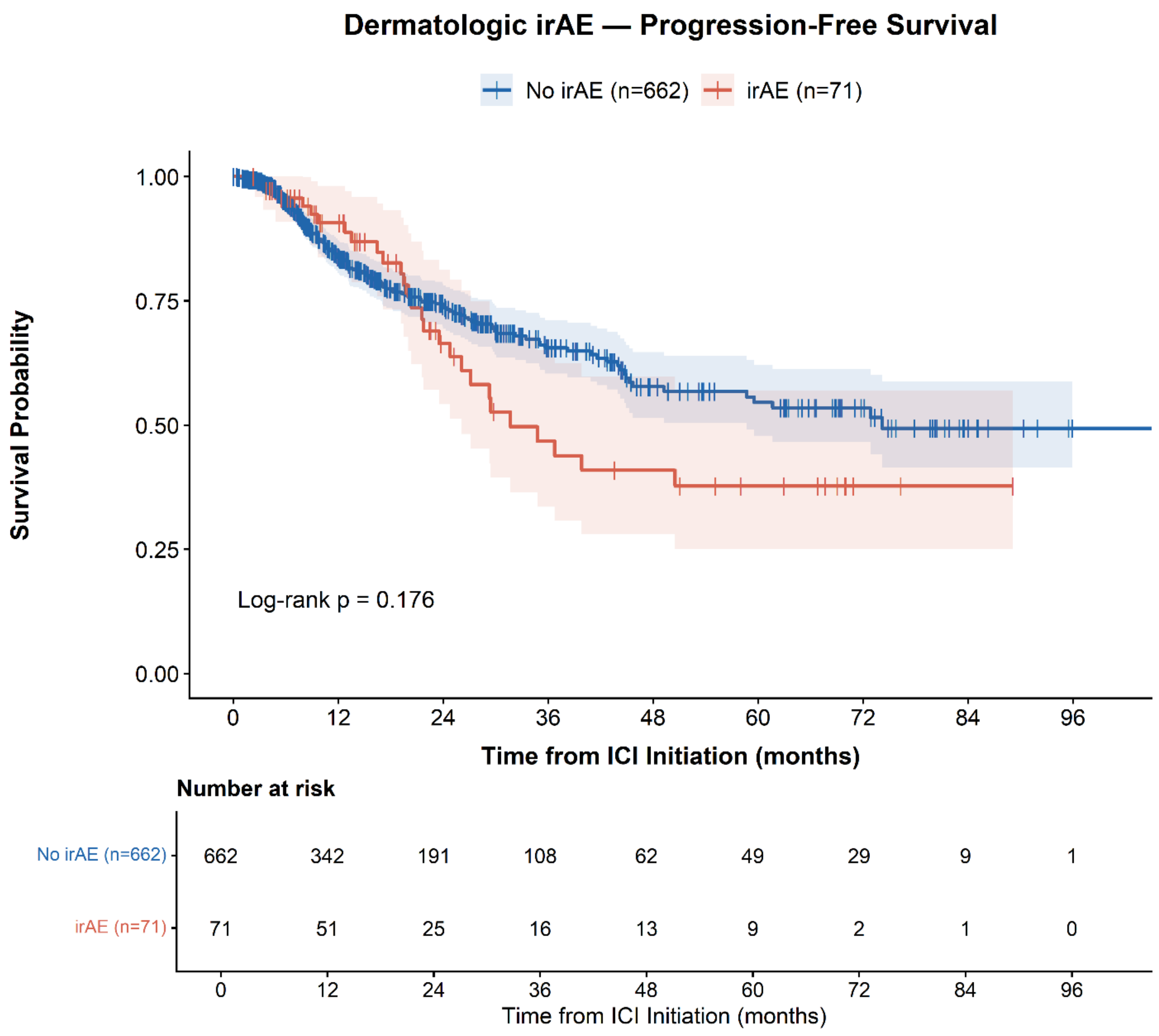
Dermatologic irAE — Progression-Free Survival
No irAE (n=662)
irAE (n=71)
Survival Probability
1.00
0.75
0.50
0.25
0.00
Log-rank p = 0.176
0
12
24
36
48
60
72
84
96
Time from ICI Initiation (months)
Number at risk
No irAE (n=662) 662 342 191 108 62 49 29 9 1
irAE (n=71) 71 51 25 16 13 9 2 1 0
0
12
24
36
48
60
72
84
96
Time from ICI Initiation (months)

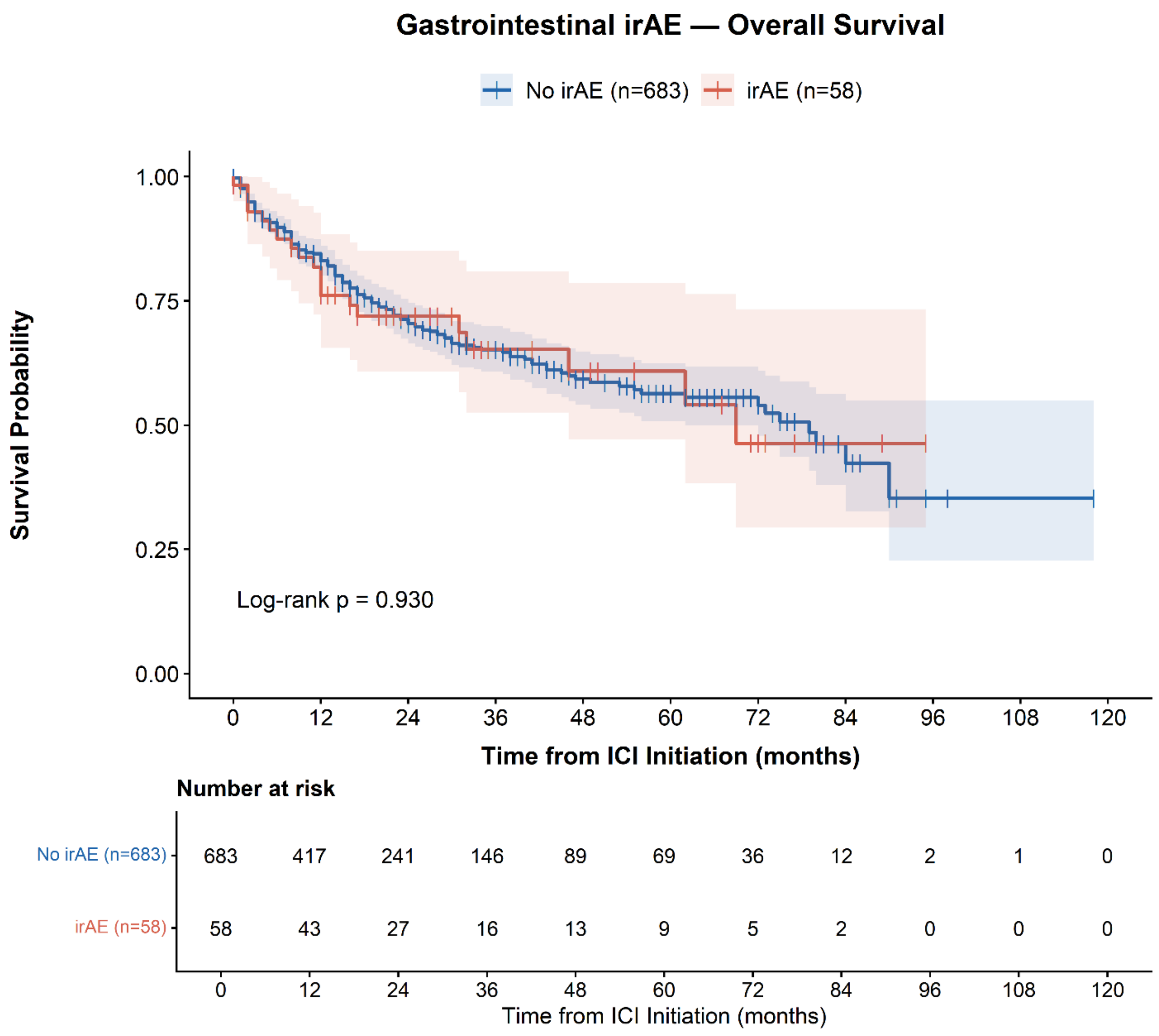

Gastrointestinal irAE — Overall Survival
No irAE (n=683)
irAE (n=58)
Survival Probability
1.00
0.75
0.50
0.25
0.00
Log-rank p = 0.930
0 12 24 36 48 60 72 84 96 108 120
Time from ICI Initiation (months)
Number at risk
No irAE (n=683) 683 417 241 146 89 69 36 12 2 1 0
irAE (n=58) 58 43 27 16 13 9 5 2 0 0 0
0 12 24 36 48 60 72 84 96 108 120
Time from ICI Initiation (months)

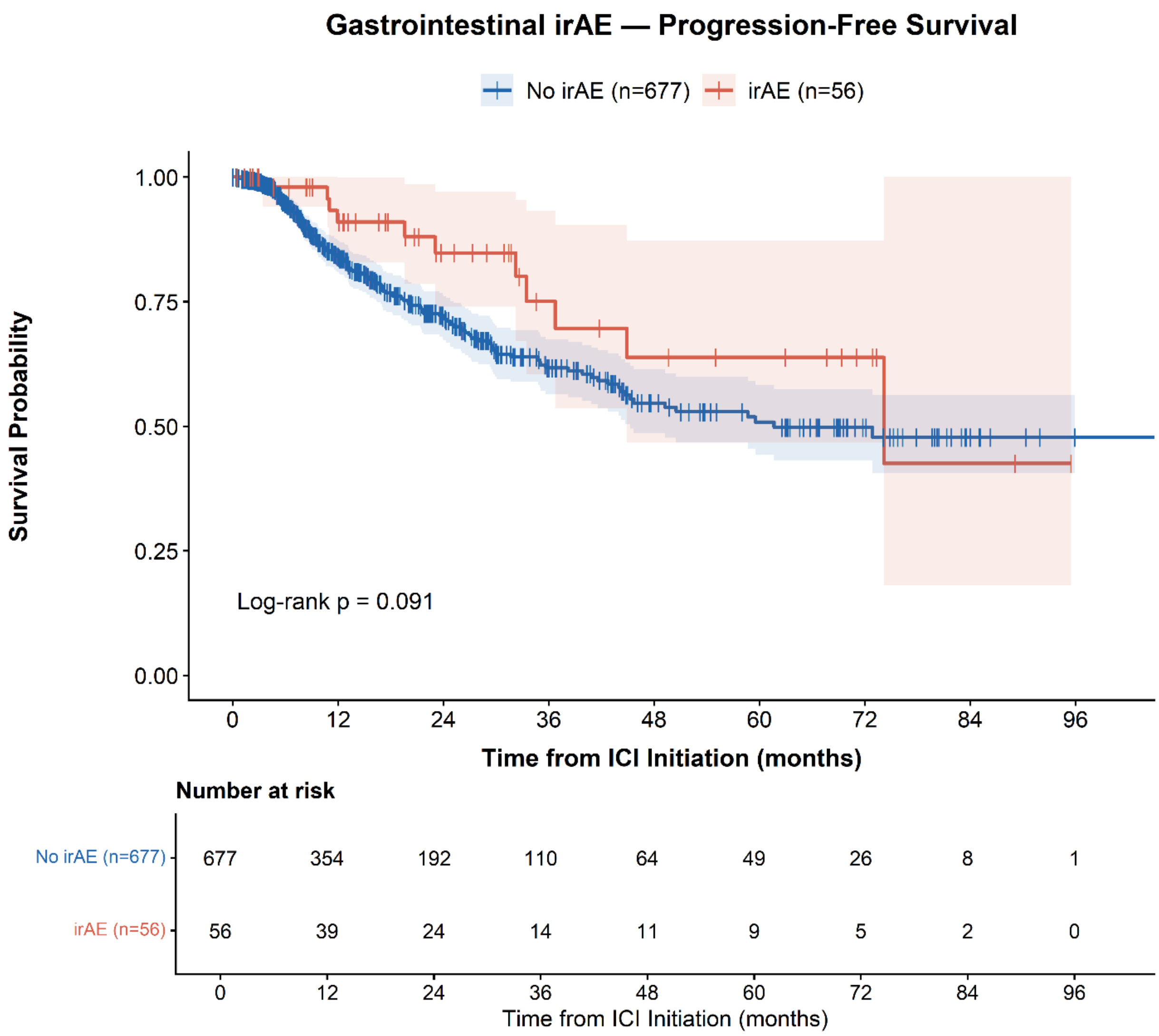
Gastrointestinal irAE — Progression-Free Survival
No irAE (n=677)
irAE (n=56)
Survival Probability
1.00
0.75
0.50
0.25
0.00
Log-rank p = 0.091
0
12
24
36
48
60
72
84
96
Time from ICI Initiation (months)
Number at risk
No irAE (n=677) 677 354 192 110 64 49 26 8 1
irAE (n=56) 56 39 24 14 11 9 5 2 0
0
12
24
36
48
60
72
84
96
Time from ICI Initiation (months)

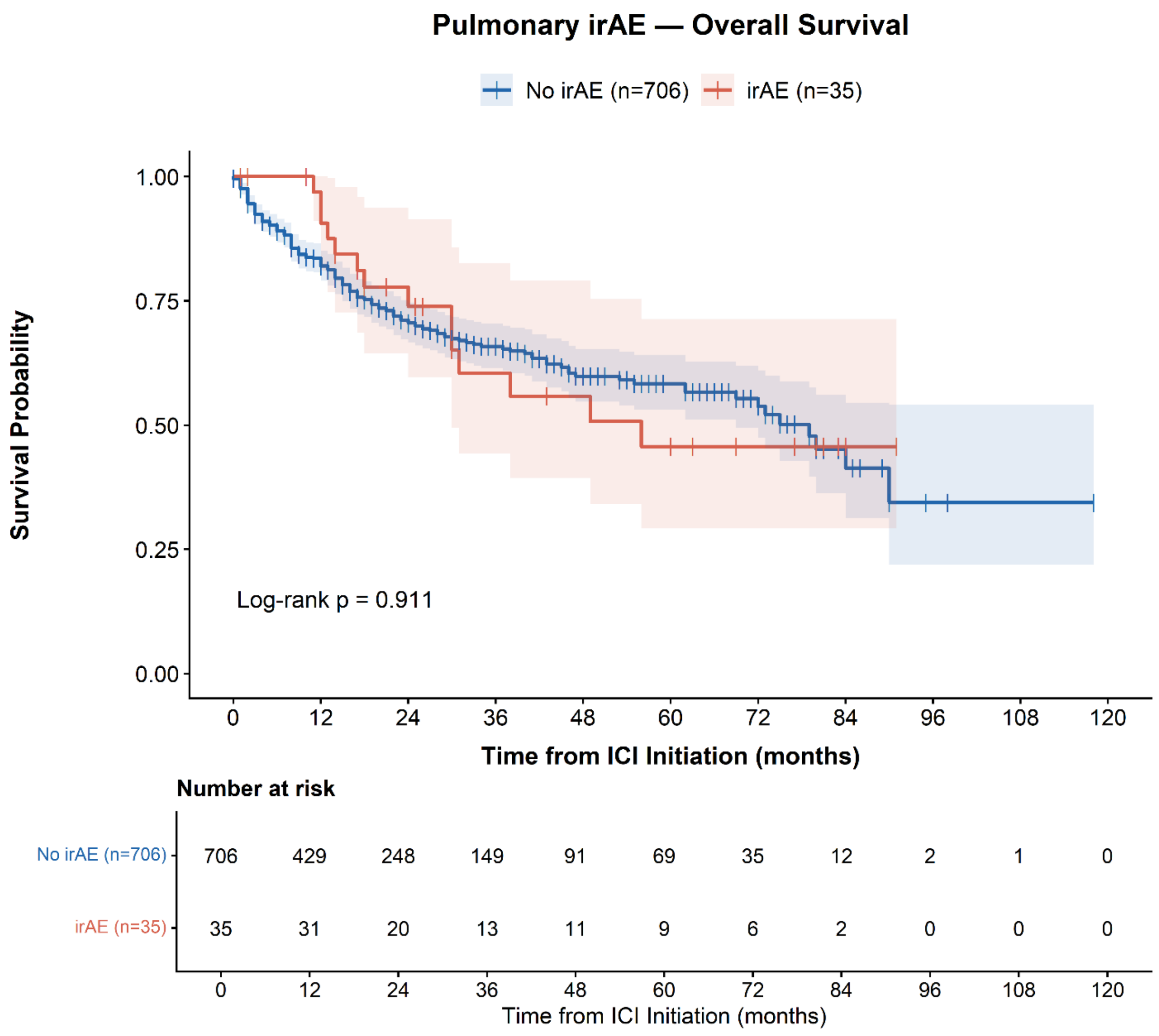

Pulmonary irAE — Overall Survival
No irAE (n=706)
irAE (n=35)
Survival Probability
1.00
0.75
0.50
0.25
0.00
Log-rank p = 0.911
0
12
24
36
48
60
72
84
96
108
120
Time from ICI Initiation (months)
Number at risk
No irAE (n=706) 706 429 248 149 91 69 35 12 2 1 0
irAE (n=35) 35 31 20 13 11 9 6 2 0 0 0
0
12
24
36
48
60
72
84
96
108
120
Time from ICI Initiation (months)

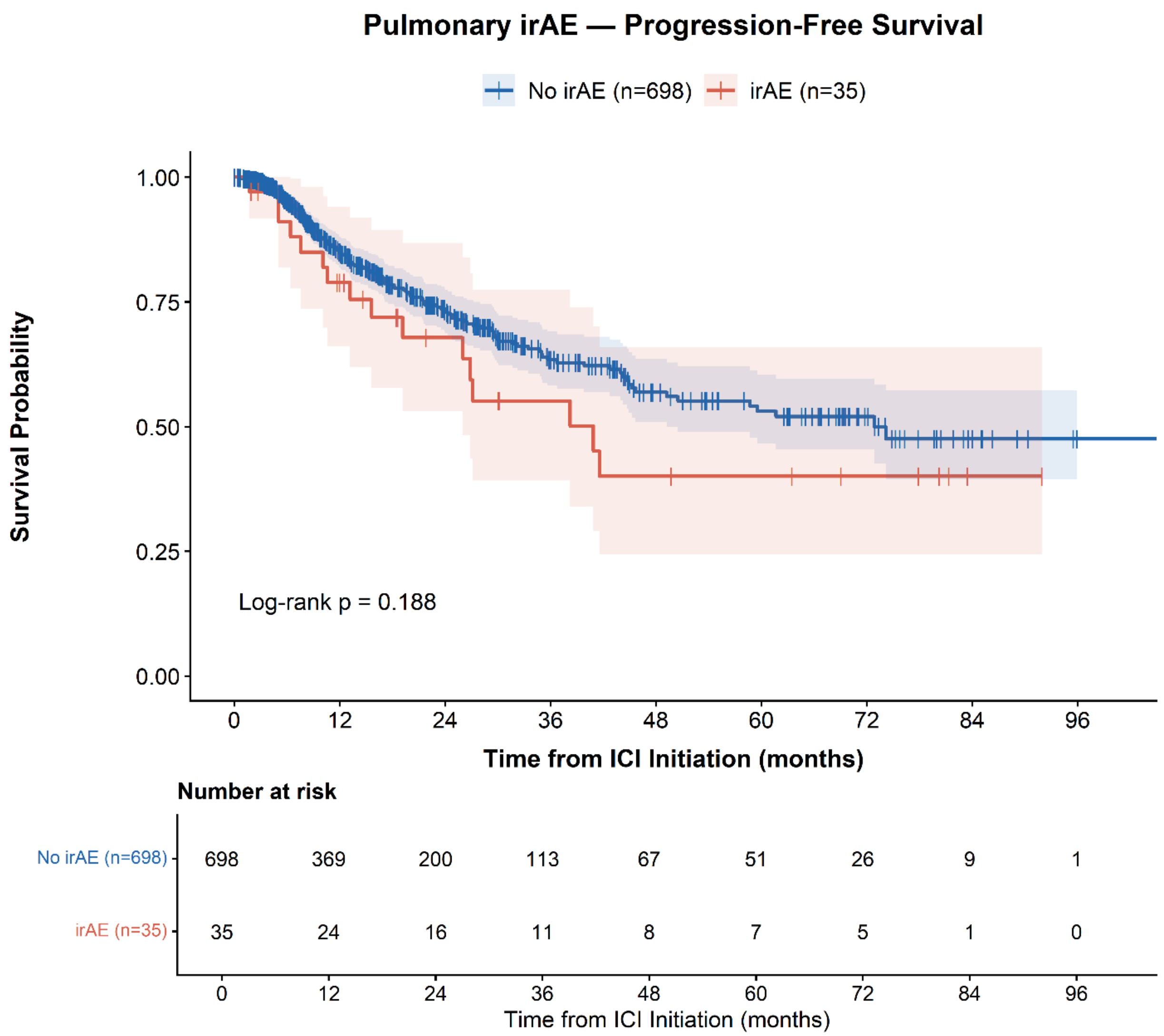
Pulmonary irAE — Progression-Free Survival
No irAE (n=698)
irAE (n=35)
Survival Probability
1.00
0.75
0.50
0.25
0.00
Log-rank p = 0.188
0
12
24
36
48
60
72
84
96
Time from ICI Initiation (months)
Number at risk
No irAE (n=698)
698
369
200
113
67
51
26
9
1
irAE (n=35)
35
24
16
11
8
7
5
1
0
Time from ICI Initiation (months)

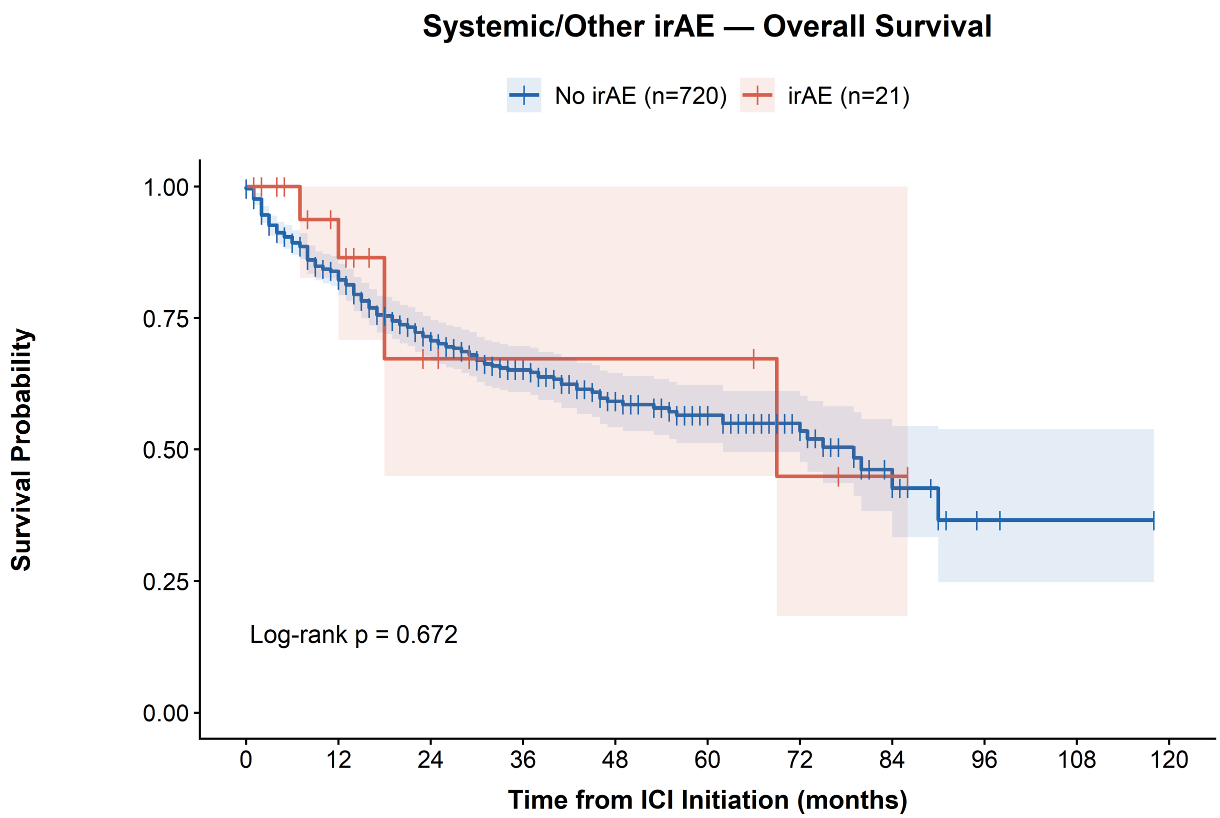

Number at risk

	0	12	24	36	48	60	72	84	96	108	120
No irAE (n=720)	720	447	262	158	98	74	39	13	2	1	0
irAE (n=21)	21	13	6	4	4	4	2	1	0	0	0

Time from ICI Initiation (months)

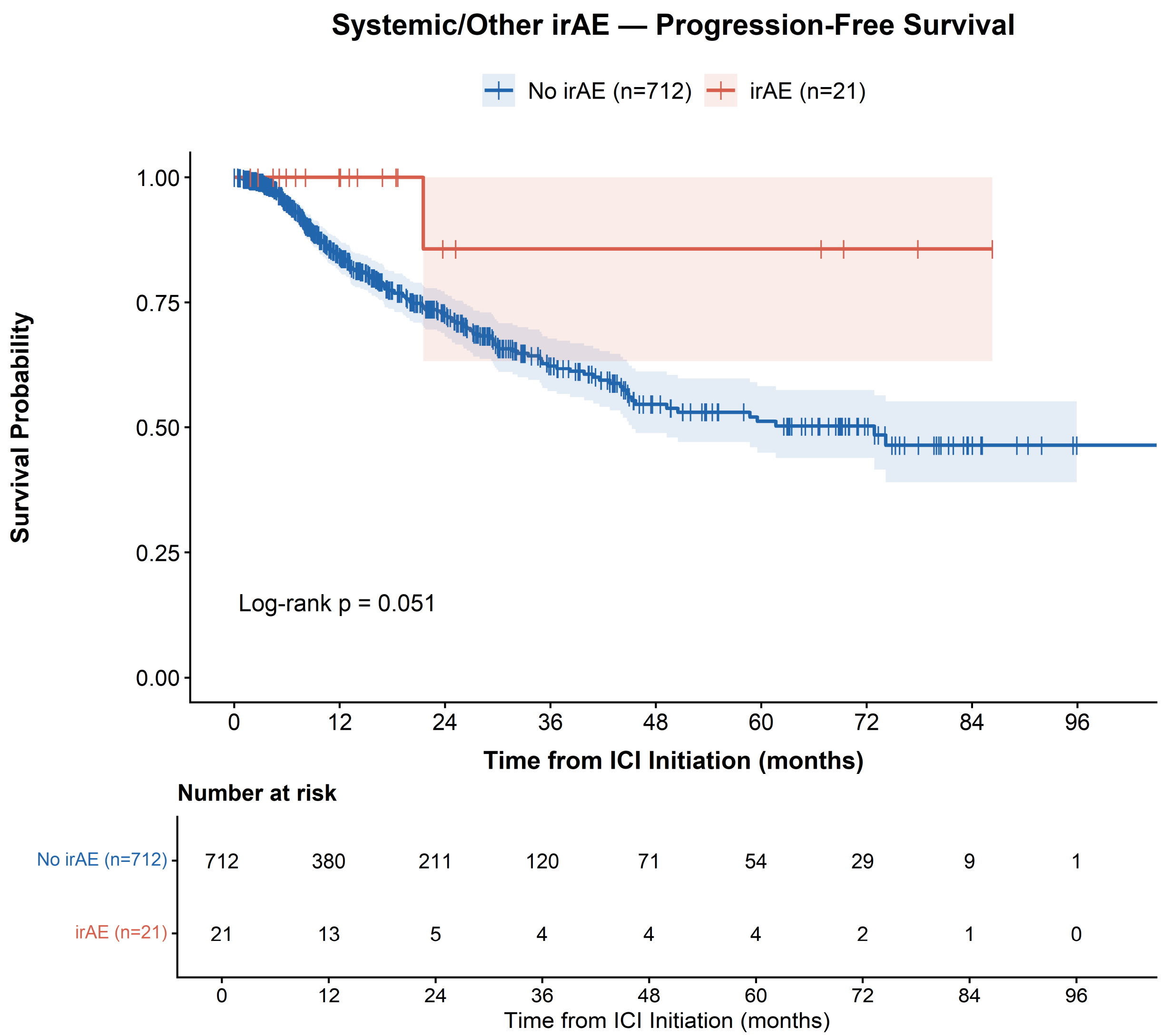

Systemic/Other irAE — Progression-Free Survival
No irAE (n=712)
irAE (n=21)
Survival Probability
1.00
0.75
0.50
0.25
0.00
Log-rank p = 0.051
0
12
24
36
48
60
72
84
96
Time from ICI Initiation (months)
Number at risk
No irAE (n=712) 712 380 211 120 71 54 29 9 1
irAE (n=21) 21 13 5 4 4 4 2 1 0
0
12
24
36
48
60
72
84
96
Time from ICI Initiation (months)

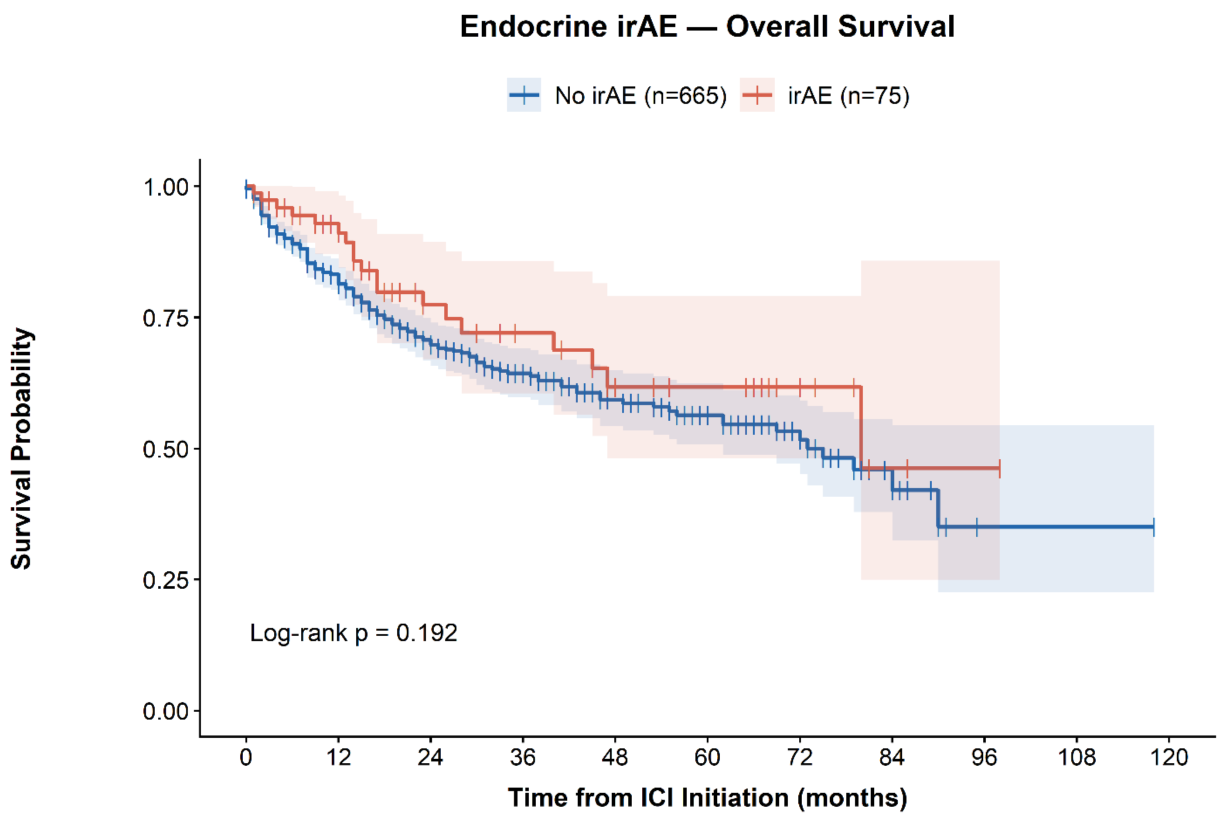

Number at risk

	0	12	24	36	48	60	72	84	96	108	120
No irAE (n=665)	665	406	238	140	86	65	33	12	1	1	0
irAE (n=75)	75	53	29	22	16	13	8	2	1	0	0

Time from ICI Initiation (months)
